# Experience-based Investigation and Co-design of Psychosis Centred Integrated Care Services for Ethnically Diverse People with Multimorbidity (CoPICS): study protocol

**DOI:** 10.1136/bmjopen-2024-084121

**Published:** 2024-02-28

**Authors:** Kamaldeep Bhui, Doreen Joseph, Nimra Khan, Tara Morrey, Roisin Mooney, Uzma Zahid, Tanya Mackay, Michael Larkin, Frank Keating, Paul McCrone, Rachel Upthegrove, Sian Lowri Griffiths, Dawn Edge, Peter A Coventry, Jason Arday, Georgina M Hosang

**Affiliations:** 1 Psychiatry, University of Oxford, Oxford, UK; 2 Queen Mary University of London, London, UK; 3 KCL, London, UK; 4 McPin Foundation, London, UK; 5 Aston University, Birmingham, UK; 6 Department of Social Work, Royal Holloway University of London, Egham, UK; 7 University of Greenwich, London, UK; 8 Department of Psychiatry, School of Psychology and College of Medical and Dental Sciences, University of Birmingham, Birmingham, UK; 9 Early Intervention Service, Forward Thinking Birmingham, Birmingham, UK; 10 University of Birmingham, Birmingham, UK; 11 University of Manchester, Manchester, UK; 12 University of York, York, UK; 13 Cambridge University, Cambridge, UK

**Keywords:** schizophrenia & psychotic disorders, chronic disease, health services

## Abstract

**Introduction:**

Ethnic minorities (also called racialised groups) are more likely to experience severe mental illness (SMI). People with SMI are more likely to experience multimorbidity (MM), making psychosis among racialised groups more likely to lead to MM, poor outcomes, disability and premature mortality.

**Methods and analysis:**

This National Institute for Health and Care Research-funded study (151887) seeks to use innovative participatory methods including photovoice and biographical narrative interviews in urban and rural areas of England to assemble experience data. These data will be subjected to polytextual thematic analysis, and alongside pictures and captions, will inform an experienced-based co-design of interventions, the implementation of which will be evaluated. There will be an economic analysis and a process evaluation of the implementation.

**Ethics and dissemination:**

This programme of work has received ethical (IRAS 322421; Newcastle North Tyneside Research Ethics Committee 23/NE/0143) and sponsor approval. The findings will be disseminated in galleries showing the creative work, as lay and academic summaries and infographics; as practice briefings for practitioners, commissioners and policy makers; peer-reviewed publications.

**Trial registration number:**

https://www.researchregistry.com/browse-the-registry%23home/registrationdetails/649c08111c037d0027b17d17/

**STRENGTHS AND LIMITATIONS OF THIS STUDY**

- The study design was informed by lived experience involvement, as will be the delivery and dissemination of findings.
- To recruit groups usually under-represented in research, we use creative, empowering and engaging participatory and biographical narrative methods.
- We will recruit ethnically diverse adults with psychosis and two or more other long-term conditions, from the National Health Service, community rural and urban sites.
- The study will take place in England.
- Implementation and evaluation of co-designed approaches will be influenced by contextual factors.

## Introduction

Black ethnic groups have a higher risk of developing severe mental illness (SMI) such as schizophrenia, and both affective and non-affective psychoses. This risk is also higher for other diverse ethnic groups.^1^ In racialised populations with SMI, physical health conditions are also more prevalent, such as asthma, cardiac disease and diabetes in South Asians, and asthma, high blood pressure and stroke in black groups.^1–4^ The National Institute for Health and Care Excellence (NICE)^5^ defines ‘multimorbidity’ as the presence of two or more long-term health conditions one of which is a physical health condition of long duration such as diabetes or infectious disease, and the other may be a further long-term physical condition or SMI. Multimorbidity can substantially reduce life expectancy.^4 6^

Among diverse ethnic populations health inequities are more prevalent.^7 8^ SMI also contributes significantly to health inequality gaps in England.^2 9^ One of the main reasons for this is that physical health needs in people with SMIs are not prioritised.^9^

Existing evidence about the prevalence of multimorbidity in racialised populations suggests that complex social determinants independently or in combination raise the risk of multimorbidity.^10^ One pertinent lens through which to view these intersecting elements is via the concept of a syndemic.^11^ A syndemic refers to a clustering of specific populations and health conditions which interact with contextual and social factors. In racialised populations at a macrolevel, this clustering may include elements such as constructions of health and healthcare, structural racism, ethnicity, environment, socio-economic status and poverty.^12–14^ At a microlevel, this may include facets like individual lived experience, childhood adversity, identity, internalised hatred, attachment and esteem.^12–14^

Such complex bio-social pathways among racialised populations appear to drive the onset of SMIs such as psychosis and other long-term physical or mental health conditions. People living with psychosis and multimorbidity face a huge burden of medical and social problems, leading to poorer health outcomes, high rates of polypharmacy, treatment burden, lower quality of life, feelings of vulnerability, disability and even premature mortality.^15–17^ Due to health-related burdens, it can be challenging for those with multimorbidity to participate in traditional forms of research.

Therefore, there is a need for integrated health and social care research and consequent interventions to attend to social determinants and socio-economic status, as these can complicate recovery and lead to inequality.^8 10^ There are issues in delivering integrated care. While there is a need to focus on both the mental and physical health of those with multimorbidity, physical and mental healthcare is not fully integrated in training professionals or across care services.^18–20^ For example, integrated service provision may be hampered by the complexity of care for patients with multimorbidity and issues in communication across physical and mental healthcare services.^18–20^ There is some research focus on what could be done in primary care, such as developing staff skills and training for primary care practitioners in cardiovascular disease prevention among those with SMI.^4^ In secondary care, suggestions for improvement include empowering SMI staff and service users to remove barriers to delivering and accessing integrated care, improved communication among healthcare professionals and better information technology to support both communication and information access.^18^

Given the higher risk of multimorbidity among racialised populations, the effect of multimorbidity on life expectancy and the priority of delivering integrated care to those with multimorbidity,^5^ this research aims to:

- support NICE guidance^5^ in improving care for multimorbidity through exploring how current care and interventions are experienced by patients, carers and healthcare professionals;
- contribute to evolving strategies that prevent the development of multiple conditions;
- understand more about causation and the role of ethnicity and health and social care in meeting the needs of people living with multimorbidity, aligning with the James Lind Alliance priorities^21^;
- facilitate the development of preventative interventions by ensuring diverse patient experiences are considered in care, especially for socially excluded groups who do not access care or find existing care unhelpful;
- draw on innovative and inclusive methods such as photovoice^22^ and experience-based co-design^23^ and recruit groups that are historically under-represented in research.

## Methods

### Design

This is a mixed methods study investigating the experiences of people living with psychosis and multimorbidities, with emphasis on those who are marginalised such as racialised communities, and service providers, commissioners, informal carers and other important stakeholders identified across the health system.

### Methodological innovations

**Figure 1:**
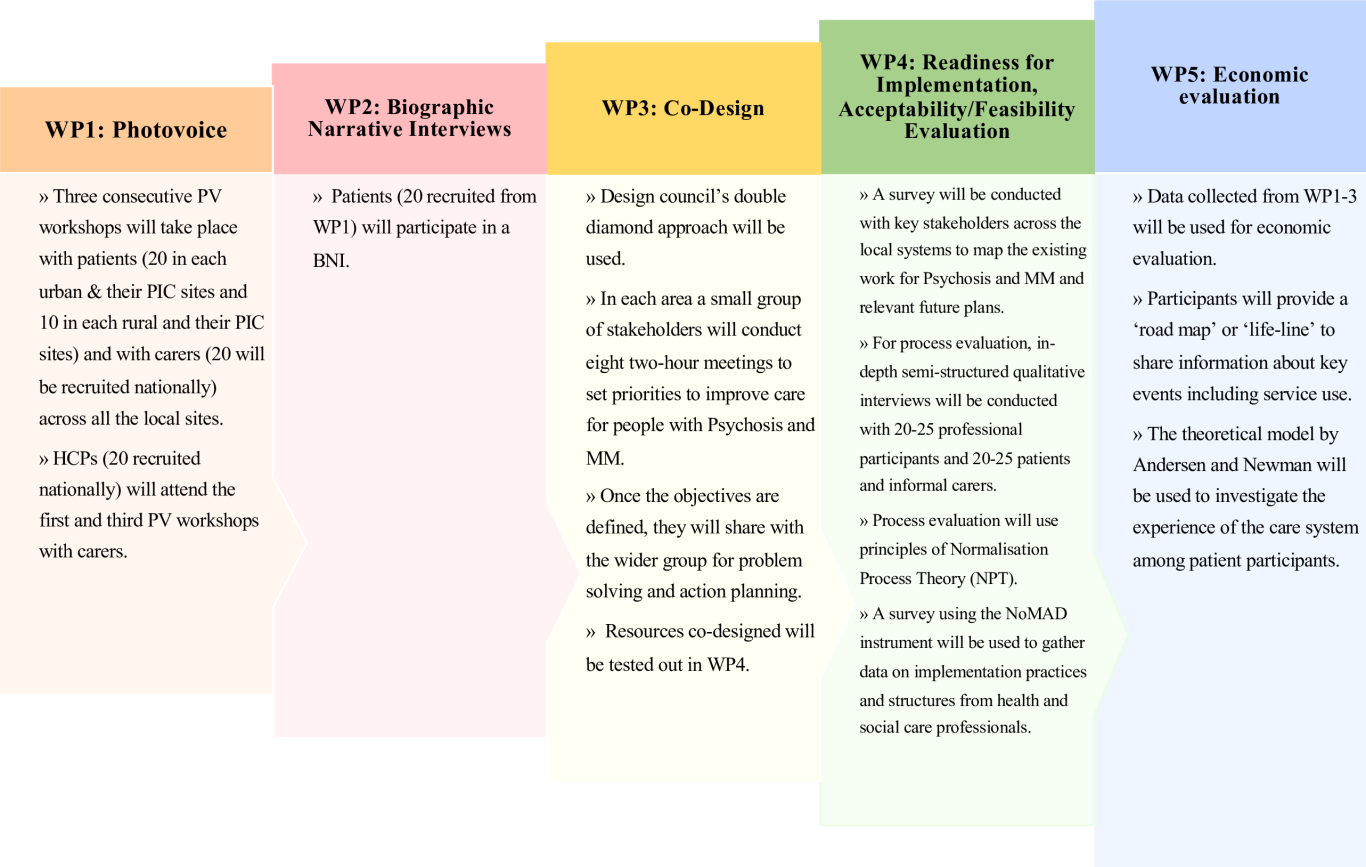
Study design. This study will be conducted in seven local systems and three participant identification centres (PIC) situated in London, Birmingham (with a PIC site in Worcestershire), Oxford (with a PIC site in Oxfordshire), Derby, Leicester, Bradford (with a PIC site in Lancashire) and Northumberland. HCP, healthcare professional; MM, multimorbidity; WP, work package; NoMAD, Normalisation MeAsure Development questionnaire.

Research studies frequently lack representation from marginalised and racialised communities.^24^ Conventional data collection methods can perpetuate power imbalances. There is a need for supportive data collection methods that engage under-represented groups.^25^ Collaborative and participatory research methods offer innovative ways to engage with communities that have been historically marginalised.^25^ Participatory research and storytelling techniques will be used to inform an experience-based co-design and process evaluation of implementation.^26^ These methods equalise power dynamics between researchers and participants, and create a secure and inclusive environment. In addition, the incorporation of art-based techniques can draw out personal experiences and cultivate potential solutions in a gentle and iterative manner. Thus, co-design approaches can include the use of creative tools including storyboards, videos, photos, clay and Lego, which have been shown to evoke deeper insights.^27^ Therefore, we will employ photovoice in work package 1 (WP1) and biographical narrative interviews (BNI) in work package 2 (WP2) to gather lived experience data (figure 1).

Photovoice (in WP1) ‘gives the lens’ to participants to take photographs and caption them to share their experiences.^28^ It is a creative methodology that enables people to express themselves and initiate critical dialogue about community issues, ultimately influencing policy.^29^ Research has shown that photovoice has positive effects on participants, leading to feelings of empowerment and social awareness about their environment. Through photovoice, participants can portray experiences that cannot be expressed with words alone, and this often leads to innovative solutions that might not have been recognised otherwise. By sharing photo stories, decision makers are sensitised to address complex health and social issues by taking action.^30^ Photovoice is particularly effective in engaging groups that are often under-represented in research, such as people with intellectual disabilities, homelessness, indigenous communities, refugees and those in mental health settings.^31–33^

WP2 involves conducting BNI with a subset of participants from WP1. The BNI methodology assumes that narrative expression can reveal both conscious concerns and unconscious individual, societal and cultural influences. It explores both the external and internal worlds with a focus on the life story, while also considering the unique historical and societal context of each case.^34^ This approach illustrates how personal and social meanings shape actions in everyday life. Additionally, oral histories through personal accounts offer valuable insights into the past and provide a voice to lived experiences.^35^ Biographical interviewing is an effective framework for ‘unpacking and deconstructing the past’.^36^ BNI is typically a multisession interview process with minimal interviewer intervention.

The information gathered from the photovoice images, their captions (WP1) and BNI-related narratives (WP2) will feed into the experience-based co-design (work package 3 (WP3)). This is a crucial opportunity for all involved to listen to each other’s perspectives and propose solutions, resulting in a workable set of options.

Work package 4 (WP4) consists of two phases. Phase I is baseline E survey to assess the landscape with regard to service provision and how this may change over the course of the research. Phase II consists of the NoMAD survey and a semi-structured interview to assess the feasibility of implementing proposed co-designed solutions or models and identify potential barriers to their broader implementation.

### Setting

This is a multicentre study and will be conducted in seven local systems and three participant identification centres (PIC) situated in London, Birmingham (with a PIC site in Worcestershire), Oxford (with a PIC site in Oxfordshire), Derby, Leicester, Braford (with a PIC site in Lancashire) and Northumberland. The chosen recruitment sites are diverse in terms of ethnic populations and geographical locations, encompassing both urban and rural areas across England.

### Participants

Throughout the study, it will be crucial to genuinely highlight the voices of marginalised populations. To achieve this, we will involve a patient and public involvement (PPI) group in all stages of this research. We will recruit 80 patients for WP1, comprising 20 from each urban area and their PIC sites as well as 10 patients from rural places and their PIC sites. We will over-recruit to account for 25% attrition. Patients are welcome to bring in carers or friends to offer them support or act as their advocate. We will collaborate with CRNs (Clinical Research Networks) and NHS Trusts to reach out to potential participants through local services, such as primary and secondary care, and social care. We will also work with non-governmental organisations, local faith communities and networks of practice and research for recruitment. We will use purposive sampling to ensure that a diverse range of experiences are captured in respect of age, ethnicity, levels of deprivation, gender and religion.^37^

We will offer support to ensure that non-English-speaking people can participate. Our team will attend board meetings, working groups and distribute information on staff intranets and throughout the local trust to raise awareness about the research. We will recruit 20 carers and 20 healthcare professionals for WP1 (photovoice) nationally. Recruitment for healthcare professionals and carers will follow the same process.

For WP2, we will recruit 20 patients who took part in WP1. For WP3 and WP4, we will work with local partners to identify key stakeholders. A small number of diverse service providers, patients and carers will be recruited for experience-based co-design (WP3). For readiness for implementation and feasibility evaluation (WP4.1), we will conduct a survey with 120 participants. This will be followed by a process evaluation (WP4.2) consisting of a NoMAD survey (n=120) and interviews (n=45) with participants, including patient participants, informal carers and healthcare professionals.

### Patient and public involvement

PPI is an essential component of good research practice that leads to more relevant and better-designed research, ensures engagement of service users and results in better outcomes.^38^ This study will employ three people with experience of mental health issues as researchers to work as part of the research team, recruiting participants, co-facilitating workshops, analysing data and sharing findings.

The entire research will be supported by a six-person Lived Experience Advisory Panel (LEAP), ensuring experiential expertise can inform the work at each stage including design, conduct and dissemination. The LEAP will be hired by McPin, a voluntary organisation that hires people with experience of mental health issues on research studies. The LEAP will meet approximately every 3 months according to the needs and progress of the study. We will design and pilot the information sheets, consent forms and data collection tools with the LEAP team.

To ensure meaningful public engagement, a person with experience of psychosis and multimorbidities is a co-investigator on the funding application, leads the LEAP team, is a member of the research team and is employed by the University of Oxford.

### WP1: photovoice procedure

In each of the local systems, three consecutive photovoice workshops will be held in a carefully selected, creative setting that fosters conversation away from clinical environments.^28^ If needed, online workshops will be available. In the first workshop, participants will be introduced to the concept of photovoice and how they can use photos and narratives to share their experiences of living with multimorbidity (psychosis and two or more long-term conditions), the complexities of care, their experiences with care, and recommendations.^28^

Participants will be provided disposable cameras and notebooks during the workshop. They will be asked to return the cameras within 2 weeks of the workshop, by meeting a member of the local study team who will post the cameras to the central study team. The notebooks will allow for participants to write notes about why they took a particular photo. If they prefer, they can also use their own phones to take photos and send them to the project email address. Participants are also welcome to use their own existing photographs that reflect their care experiences. The project team will consider other creative materials as well, such as drawings, poetry, music and videos.

In the second workshop, participants will be presented with their images and will be guided through a reflective process (prompts presented in box 1) to generate narratives and captions for a minimum of 3–5 selected images (resulting in a total of 180–300 items). The second workshop will be a drop-in session lasting at least 2 hours, and can be attended at any time within a 6–8 hours time window. The third workshop will take place a week after the second workshop, and will provide an opportunity for participants to share their images and experiences with each other.^39^ The third workshop will be audio recorded and transcribed verbatim. There will be no topic guide, but the conversation will be guided by which images participants choose to share, and guided by two overarching questions ‘what could be different?’ and ‘what made a difference?’

Box 1 **Prompt questions used to elicit participant experiences**

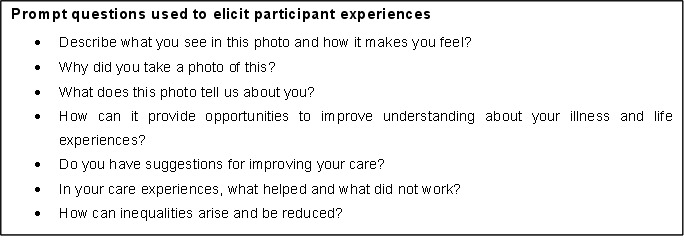

We will also conduct three photovoice workshops for 20 carers of those with psychosis and multimorbidities (generating 60–100 images and narrative captions). We will invite 20 professionals responsible for providing care for individuals with psychosis and multimorbidity including commissioners, general practitioners, physicians, psychiatrists, psychologists, social workers and nurses. Selection criteria for photovoice workshops is presented in table 1. The professionals will be invited to attend two workshops—an introductory one and a reflective one. We anticipate that both carers and professionals will be from the same regions as the individuals living with psychosis and multimorbidity.

**Table 1:** Photovoice workshops selection criteria

Inclusion criteria	Exclusion criteria
**For patient participants**	
Over the age of 18 years	Acute distress or immediate health problems that must be prioritised (eg, suicidal thinking/intent, urgent need for medical intervention or series of treatments)
Index diagnosis of psychosis and at least two of the following conditions known to lead to premature death: diabetes, cardiorespiratory, kidney and liver disease	People with only psychosis and a common mental disorder, such as anxiety or mood disorders
We will seek to ensure representation of those facing most inequality; for example, black Caribbean and black African, as well as Indian, Pakistani and Bangladeshi groups of men and women; and mixed groups, Eastern Europeans, asylum seekers/refugees and white British identifying people	
Willing and able to provide consent	
**For carers**	
Over the age of 18 years	Formal carers
Informal caring responsibilities for someone who meets the patient selection criteria	Unable to provide consent
**For healthcare professionals**	
Over the age of 18 years	>6 months experience of working with people with psychosis
Working in any capacity in any of the systems that work with people with psychosis (such as social work, psychiatry, psychology, general practice and nursing)	Unable to provide consent

### WP2: biographic narrative interviews procedure

The interviews, ideally, will be conducted in person, with much flexibility around duration and ways in which people can respond (written, verbal, recorded, etc).^34^

These interviews can last up to a maximum of 6 hours. However, they do not need to be done in one sitting, rather on separate days, or in short bursts of 20 min or an hour, by written or oral responses to prompt questions, or a combination of the above; the exact approach will be personalised, based on individual preferences. Since the interview is about the participant’s story-telling, it is crucial not to rush them but rather enable their stories to unfold in the way they would like to tell them.^35^ Notably, these interviews are not structured, interrogative, inflexible interviews, but the participant is in charge of their story and the way it is told; therefore, these approaches are enjoyed, and do not place the same burden on participants as surveys.

Both peer researchers and research assistants will conduct and analyse interviews. We will ensure that adequate training and support in BNI is undertaken. We will co-produce a distress protocol during the training to ensure that the relevant processes are in place, and that those conducting the interviews are confident in implementing this protocol.

### WP3: co-design procedure

The study will follow the Design Council’s Double Diamond approach, which involves four stages: discover, define, develop and deliver.^40^ This process helps to gain a better understanding of the issue at hand and take focused action. The aim of these workshops will be to co-design resources and consider their acceptability, feasibility, barriers to implementation and mechanisms of effect to improve care experiences of people living with psychosis and multimorbidity.

#### Discover

This phase allows a comprehensive understanding of the issue by engaging with those directly impacted by it. This will be undertaken in WP1 (photovoice) and 2 (BNI). The outputs from WP1 and WP2 will be analysed and curated to be presented to the core group in a series of priority setting meetings (3–6 of these) to identify key ‘touch points’ that characterise positive and negative experiences.

#### Define and develop

The information gathered in the discover phase will be used to define the issue. A range of people will be consulted for their views about the problem and solutions will be co-designed. To establish trust, the same key participants—those who have lived experience of the complex phenomenon being examined, as well as those involved in priority setting—will be involved in these phases. The process will entail eight meetings, each lasting 2 hours.

There will be a series of group meetings, both in-person and online. The initial meetings will involve sharing preliminary ideas and experiences among the core members, followed by identifying patterns and priorities. Once the core group has established their objectives and priorities, they will present these to the wider group for final problem-solving and action-planning. This approach aligns with the psychosocial process employed in experience-based co-design, which fosters a sense of psychological safety within a group who share similar perspectives, when people are first expressing ideas and exploring how they feel about them.

#### Deliver

The resources co-designed in the design and develop phases will be implemented and improved to adjust to real-world situations. This phase will be located in WP4 as part of the acceptability and feasibility work and a process evaluation of resources within sites. Selection criteria for co-design workshops is presented in table 2.

**Table 2:** Experience-based co-design workshops selection criteria

Inclusion criteria	Exclusion criteria
People with lived experience of receiving care or delivering care in the relevant systems, and people with the power to make decisions and implement changes in the system	No lived experience of psychosis or MM or working with people with psychosis or MM
Willing and able to provide consent	

MM, multimorbidity.

### WP4: evaluation of readiness for implementation, acceptability and feasibility

#### Phase I: Baseline survey

This will be conducted at T1 (before WP1), T2 (after WP2) and T3 (after WP3). At the beginning of the research (T1), we will survey each of the participating regions, including key decision-making stakeholders across all sectors in an ICS (Integrated Care Systems)to establish: plans for integration of care and services; existing work or planned work on psychosis and multimorbidity; the priority given to psychosis and multimorbidity and the relevance of ethnicity in their work; plans over the coming 1,3,5 years; networks of importance and key leaders in the localities that we should contact; invitation to participate in an ongoing network including dissemination of the research and innovations and sharing best practice. The survey will be short, 5–10 questions with Likert scales and open text boxes. At T2 and T3 the survey will be repeated. The objective is to look at the broad brushstrokes in relation to any shifts in priorities and to sensitise the research team towards the process evaluation.

We will survey up to 120 ICS leaders and managers and practitioners in participating localities as possible, setting a minimum of 10 and up to 30 key stakeholders identified with our co-investigators and project partners from each locality. We will repeat this after WP2, and after WP3, to evaluate receptivity to implement innovations, any changes in the action plans on psychosis and multimorbidity in ethnically diverse samples. We anticipate participatory work itself has an impact, so we will be able to gauge to what extent this happens, and variations over time in a descriptive way to inform an assessment of readiness to change and barriers and facilitators. Participants may be invited back for the process evaluation in the last 6 months of the project after sharing emergent findings.

#### Phase II: process evaluation

A process evaluation will be conducted using the Normalisation Process Theory (NPT).^41^ NPT is a conceptual framework based on four generative mechanisms that underpin the implementation of complex interventions: coherence (what is the work to be done?); cognitive participation (participants have to buy-in to the work); collective action (what work has to be done to enact and enable new practices?) and reflexive monitoring (what work can be done to help appraise new practices?). We will use in-depth semi-structured qualitative interviews with 20–25 professional participants, including decision-makers, commissioners and health professionals who have used the co-designed resources in hospital, primary and community care settings. We will also interview up to 20–25 patients and informal carers. Selection criteria for process evaluation is presented in table 3. Patients and carers will be interviewed separately by default, but may choose to be interviewed together. In such cases, interviews will be conducted in a dyadic fashion. Patient participants will be purposively sampled for maximum variation in relation to age, recruitment site, gender, socio-economic background and mental health status. Furthermore, health and social care professionals will be surveyed (n=120) using the NoMAD instrument to gather data on implementation practices and structures.^42^ The survey questions will be tailored to identify the potential impact of the co-designed resources and whether they will be integrated into routine usage. This will help in determining the usefulness of the resources and inform their future use.

**Table 3:** Process evaluation survey and selection criteria for conducting interviews

Inclusion criteria	Exclusion criteria
**For survey**	
Participants will be healthcare professionals working in primary care practices, secondary care mental health services and/or employees of integrated care systems in clinical and non-clinical roles	Those in non-professional or non-NHS facing job roles will be excluded from the survey
Willing and able to provide consent	
**For interviews**	
Eligibility criteria for patient participants will be the same as WP1	Exclusion criteria for patient participants will be the same as WP1
Eligibility criteria for carers will be the same as WP1	Exclusion criteria for patient participants will be the same as WP1
Healthcare professionals will be eligible if they are employed in primary care or secondary care mental health services in the same geographic footprint of participating ICS	Healthcare professionals working outside the participating ICS case study regions will be excluded

WP, work package.

### WP5: economic evaluation

Data collected from WP1–3 will be used for economic evaluation. We will ask patient participants to provide information about their experiences of healthcare services before and after their diagnosis of psychosis. In WP1 during the second workshop, we will ask participants to provide a ‘road map’ or ‘life-line’ to help them recall events. We will use the theoretical model established by Andersen and Newman to investigate the experience of the care system among patient participants.^43^ This model identifies three key types of factors that could influence the use of healthcare services: predisposing features (eg, age, sex, ethnicity, previous illnesses), enabling features (eg, income, access to care, employment) and needs (eg, symptoms, diagnosis, functioning).^17^ Furthermore, participants will be encouraged to indicate any care they would have preferred to receive but did not, as well as suggest reasons or barriers for why this was the case. The removal of these barriers may have cost implications and could also impact outcomes, which we will explore using economic modelling methods.

### Analysis

Data across work packages will be collected in the form of photographic images and captions, written or other reflective accounts of experience, such as drawings and poetry, biographical narratives and group discussions, surveys. Data will also be collected via qualitative interviews and a baseline survey concerning available services and the NoMAD survey for a process evaluation. The Client Service Receipt Inventory (CSRI) will be used to record the use of health and social care services.

WP1 photovoice will be analysed using a combination of framework analysis^44^ and polytextual thematic analysis^45 46^ to both organise and understand the data. The work package will render three levels of data: demographic data, images and captions and transcripts from the discussion of the images among participants from the third workshop. The following eight steps of polytextual thematic analysis will be used to analyse the data^46^: step 1—understanding the sample, step 2—organising the data, step 3—individual case analysis, step 4—exploring interactions, step 5—combining the individual and group analyses, step 6—theme generation, step 7—polytextual thematic analysis, 8—dissemination.^46^

WP2 BNI will be audio recorded and transcribed verbatim. The transcripts will be imported to MAXQDA. Thematic analysis^47^ will be used to code the data, and from this, themes will be generated. The resulting themes will be considered alongside the analysis from WP1 to look for points of both similarity and contention. Peer researchers will be supported to also code transcripts and will be part of the team discussions to consider the iteration of higher order themes.

WP3 experience-based co-design workshops will be audio recorded and transcribed verbatim. The purpose is to identify the context and reasons for decisions about co-design, to address indicated and agreed touchpoints. There may also be realisations that some groups are not going to be well served if a particular design or set of priorities are proposed. The narrative accounts will be described to set out such decisions, rather than needing a formal qualitative analysis process. We will ensure all respective views and positions are represented and the narrowing of options towards consensus is evident. Those participating in the co-design workshops will be given an opportunity to review the transcripts and summary documents.

WP4 is a process evaluation. which has two phases. Phase I is a baseline e survey that will take place at T1 (prior to WP1), T2 (after WP2) and T3 (after WP3) and will be analysed descriptively using means, SD and 95% CIs. Inductive content analysis will be used for categorising the free-text responses. Responses will be descriptively coded and reviewed to identify representative phrases that are characteristic of or overlap with professional groupings.

Phase II of WP4 takes place after WP3 and data will be collected via in-depth semi-structured interviews and the NoMAD survey.^48^ All interview data will be transcribed verbatim and analysed thematically. An initial thematic coding framework will be developed and checked. Codes in each interview will be examined across individual transcripts as well as the entire dataset and allocated to the framework. Using aspects of the constant comparative analysis method broader categories using linked codes will be developed across interviews. Analysis will be guided by items of NPT to structure patients’, carers’ and professionals’ views about acceptability and implementation readiness of the intervention.

The NoMAD survey tool includes 20 items that reflect the 4 core NPT constructs and 3 items to capture data about participants’ views about the likelihood that an intervention can become a routine part of their work. The 20 items that compare responses across the 4 NPT constructs are scored using a 5-point Likert scale to indicate the level of agreement, where 1=strongly agree, 3=neutral and 5=strongly disagree. The additional three generic questions about suitability of the intervention for routine practice are scored using a response scale from 0 to 10, with 0=not at all; 5=somewhat and 10=completely. We will analyse NoMAD responses descriptively, reporting absolute and relative frequencies, along with means and SD and 95% CIs. Where items are negatively phrased responses will be inversely scored. Total scores will not be calculated.

WP5 health economics is using the CSRI to record the use of health and social care services. These data will be combined with appropriate unit cost information to calculate care costs. Regression models will be used to identify significant demographic and clinical predictors of cost. The potential cost of services not received but perceived as important by participants will also be calculated. Decision modelling will examine the impact of removing care barriers on costs.

### Ethics and dissemination

The study has received a favourable ethics opinion from the Newcastle North Tyneside Research Ethics Committee (reference number: 23/NE/0143; IRAS 322421). All participants will be provided with detailed information about what participation entails through a participant information sheet, as well as through verbal explanation to ensure full understanding.^49^ Only individuals with the capacity to consent will be included in the study, and informed consent will be obtained at each phase of the research. Participants will be informed that they are free to leave the study at any time without having to provide any reasons. Participants will be assured that they will not face any negative consequences regarding their care. Given that individuals with multimorbidity and psychosis from marginalised communities are often more vulnerable, the research team and local systems will take measures to protect, empower and provide necessary support to participants throughout the research process.^16^ This will prevent any potential power imbalances and foster a relationship of trust between researchers and participants. Furthermore, the research methodologies employed seek to minimise stress, provide opportunities for participants to express their artistic abilities and do not necessitate the verbalisation of emotionally challenging experiences.^50^ The confidentiality and anonymity of participants’ personal information shall be strictly maintained. The photos and captions will not reveal the identities of the participants unless they make a request for disclosure.

This project will prioritise knowledge exchange and collaborative working. We will take care to disseminate the outputs so the participants do not feel disempowered or lose ownership. To create awareness, we will use online resources such as social media platforms like Twitter, Instagram and TikTok, as well as a dedicated website and monthly newsletters. Photos and captions taken with WP1 will be shared through exhibitions across local sites to showcase the experiences of individuals with psychosis and multimorbidity. The hope is that these shared experiences will inspire service providers and policy makers to work towards improving services. Furthermore, academic publications will be authored along with partners to further the discussions about using creative methodologies and reducing disparities among diverse groups.

## Supplementary Material

Reviewer comments

Author's manuscript
