# Supplementary material for: Experience-based Investigation and Co-design of Psychosis Centred Integrated Care Services for Ethnically Diverse People with Multimorbidity (CoPICS): study protocol
Source: BMJ Open. 2024 Feb 28;14(2):e084121. doi: 10.1136/bmjopen-2024-084121 (PMC10910695; doi:10.1136/bmjopen-2024-084121)
Supplement: Author's manuscript [file bmjopen-2024-084121.draft_revisions.pdf]

BMJ Open is committed to open peer review. As part of this commitment we make the peer review history of every article we publish publicly available.

When an article is published we post the peer reviewers' comments and the authors' responses online. We also post the versions of the paper that were used during peer review. These are the versions that the peer review comments apply to.

The versions of the paper that follow are the versions that were submitted during the peer review process. They are not the versions of record or the final published versions. They should not be cited or distributed as the published version of this manuscript.

BMJ Open is an open access journal and the full, final, typeset and author-corrected version of record of the manuscript is available on our site with no access controls, subscription charges or pay-per-view fees (http://bmjopen.bmj.com).

If you have any questions on BMJ Open's open peer review process please email info.bmjopen@bmj.com

BMJ Open

Experience-based Investigation and Co-design of Psychosis Centred Integrated Care Services for Ethnically Diverse People with Multimorbidity (CoPICS): Study Protocol.

Journal:	*BMJ Open*
Manuscript ID	bmjopen-2024-084121
Article Type:	Protocol
Date Submitted by the Author:	09-Jan-2024

Complete List of Authors:	Bhui , Kamaldeep; University of Oxford, Psychiatry ; Queen Mary University of London<br>Joseph , Doreen ; University of Oxford, Psychiatry<br>Khan, Nimra; University of Oxford, Psychiatry<br>Morrey, Tara; University of Oxford, Psychiatry<br>Mooney, Roisin; University of Oxford, Psychiatry<br>Zahid, Uzma; KCL<br>Mackay, Tanya; McPin Foundation<br>Larkin, Michael; Aston University<br>Keating, Frank; Royal Holloway University of London, Department of Social Work<br>McCrone, Paul; University of Greenwich<br>Upthegrove, Rachel; University of Birmingham, Department of Psychiatry, School of Psychology and College of Medical and Dental Sciences; Forward Thinking Birmingham, Early Intervention Service<br>Griffiths, Sian Lowri; University of Birmingham<br>Edge, Dawn; Univ Manchester<br>Coventry, Peter; University of York,<br>Arday, Jason; Cambridge University<br>Hosang, Georgina M.; Queen Mary University of London

Keywords:	Schizophrenia & psychotic disorders < PSYCHIATRY, Chronic Disease, Health Services

SCHOLARONE™
Manuscripts

Word count:

Main text 4693

Abstract 231

Experience-based Investigation and Co-design of Psychosis Centred Integrated Care Services for Ethnically Diverse People with Multimorbidity (CoPICS): Study Protocol.

*Corresponding author Prof. Kamaldeep Bhui, Dept of Psychiatry, Warneford Hospital, Oxford OX3 7JX.

University of Oxford
*Kamaldeep Bhui, professor
Doreen Joseph, lived experience expert
Nimra Khan, post-doctoral researcher
Tara Morrey, post-doctoral researcher
Roisin Mooney, post-doctoral researcher

King's College London
Uzma Zahid, Research Fellow

The McPin Foundation
Tanya Mackay, lived experience lead

Aston University, Birmingham
Michael Larkin, professor

Royal Holloway University of London
Frank Keating, professor

Greenwich University
Paul McCrone, professor

University of Birmingham
Rachel Upthegrove, professor
Sian Griffiths, research fellow

University of Manchester
Dawn Edge, professor

University of York
Peter Coventry, professor

University of Cambridge
Jason Arday, professor

Queen Mary University of London,
Georgina Hosang, Reader

For peer review only

Abstract

Introduction: Ethnic minorities (also called racialised groups), are more likely to experience severe mental illness (SMI). People with SMI are more likely to experience multimorbidity (MM), making psychosis amongst racialised groups more likely to lead to MM, poor outcomes, disability, and premature mortality.

Methods and analysis: This NIHR funded study [151887] seeks to use innovative participatory methods including photovoice and biographical narrative interviews in urban and rural areas of England to assemble experience data. This data will be subjected to polytextual thematic analysis, and alongside pictures and captions, will inform an experienced based co-design of interventions, the implementation of which will be evaluated. There will be an economic analysis and a process evaluation of the implementation.

Ethics and dissemination: This programme of work has received ethical (IRAS 322421) and sponsor approval. The findings will be disseminated in galleries showing the creative work, as lay and academic summaries and infographics; as practice briefings for practitioners, commissioners, and policy makers; peer reviewed publications.

Strengths and limitations: The design of the study, and delivery, and dissemination are all informed by lived experience. The study will recruit those under-represented in reach and use creative, empowering, and engaging participatory methods to do so. The study takes place in England and will recruit adults with psychosis and at least two other long term health conditions from NHS and community sites in rural and urban areas of England.

For peer review only

Introduction

Black ethnic groups have a higher risk of developing severe mental illness (SMI) such as schizophrenia, and both affective and non-affective psychoses. This risk is also higher for other diverse ethnic groups [1]. In racialised populations with SMI physical health conditions are also more prevalent, such as asthma, cardiac disease and diabetes in South Asians, and asthma, high blood pressure and stroke in Black groups [1-4]. NICE [5] defines 'multimorbidity' as the presence of two or more long term health conditions one of which is a physical health condition of long duration such as diabetes or infectious disease, and the other may be a further long-term physical condition or SMI. Multimorbidity can substantially reduce life expectancy [4, 6].

Amongst diverse ethnic populations health inequities are more prevalent [7, 8]. SMI also contributes significantly to health inequality gaps in England [2, 9]. One of the main reasons for this, is that physical health needs in people with SMI's are not prioritised [9].

Existing evidence about the prevalence of multimorbidity in racialised populations suggests that complex social determinants independently or in combination raise the risk of multimorbidity [10]. One pertinent lens through which to view these intersecting elements is via the concept of a syndemic [11]. A syndemic refers to a clustering of specific populations and health conditions which interact with contextual and social factors. In racialised populations at a macro level this clustering may include elements such as: constructions of health and health care, structural racism, ethnicity, environment, socio-economic status and poverty [12-14]. At a micro level this may include facets like individual lived experience, childhood adversity, identity, internalised hatred, attachment and esteem [12-14].

For peer review only

Such complex bio-social pathways amongst racialised populations appear to drive the onset of SMI's such as psychosis and other long-term physical or mental health conditions. People living with psychosis and MM face a huge burden of medical and social problems, leading to poorer health outcomes, high rates of polypharmacy, treatment burden, lower quality of life, feelings of vulnerability, disability, and even premature mortality [15-17]. Due to health-related burdens, it can be challenging for those with MM to participate in traditional forms of research.

Therefore, there is a need for integrated health and social care research and consequent interventions to attend to social determinants and socio-economic status, as these can complicate recovery and lead to inequality [8, 10]. There are issues in delivering integrated care. Whilst there is a need to focus on both the mental and physical health of those with multimorbidity, physical and mental health care is not fully integrated in training professionals or across care services [18-20]. For example, integrated service provision may be hampered by the complexity of care for patients with multimorbidity and issues in communication across physical and mental healthcare services [18-20]. There is some research focus on what could be done in primary care, such as developing staff skills and training for primary care practitioners in cardio-vascular disease prevention amongst those with SMI [4]. In secondary care suggestions for improvement include empowering SMI staff and service users to remove barriers to delivering and accessing integrated care, improved communication amongst healthcare professionals and better information technology to support both communication and information access [18].

Given the higher risk of multimorbidity amongst racialised populations, the effect of multimorbidity on life expectancy, and the priority of delivering integrated care to those with multimorbidity [5] this research aims to:

For peer review only - http://bmjopen.bmj.com/site/about/guidelines.xhtml

- support NICE guidance [5] in improving care for multimorbidity through exploring how current care and interventions are experienced by patients, carers and health care professionals.
- contribute to evolving strategies that prevent the development of multiple conditions
- understand more about causation and the role of ethnicity and health and social care in meeting the needs of people living with multimorbidity, aligning with the James Lind Alliance priorities [21].
- facilitate the development of preventative interventions by ensuring diverse patient experiences are considered in care, especially for socially excluded groups who do not access care or find existing care unhelpful.
- draw upon innovative and inclusive methods such as photovoice [22] and experience-based co-design [23] and recruit groups that are historically under-represented in research.

Methods

Design

Participatory research and storytelling techniques will be used to inform an experience-based co-design and process evaluation of implementation [26]. This is a mixed methods study investigating the experiences of people living with psychosis and multimorbidities (MM), with emphasis on those who are marginalised such as racialised communities, and service providers, commissioners, informal carers, and other important stakeholders identified across the health system.

Figure 1: Study design

For peer review only - http://bmjopen.bmj.com/site/about/guidelines.xhtml

Methodological Innovations

Research studies frequently lack representation from marginalised and racialised communities [24]. Conventional data collection methods can perpetuate power imbalances. There is a need for supportive data collection methods that engage underrepresented groups [25]. Collaborative and participatory research methods offer innovative ways to engage with communities that have been historically marginalised [25]. These methods equalise power dynamics between researchers and participants, and create a secure and inclusive environment. In addition, the incorporation of art-based techniques can draw out personal experiences and cultivate potential solutions in a gentle and iterative manner. Thus, co-design approaches can include the use of creative tools including storyboards, videos, photos, clay, and Lego, which have been shown to evoke deeper insights [27]. Therefore, we will employ Photovoice (PV) in work package 1 (WP1) and Biographical Narrative Interviews (BNI) in work package 2 (WP2) to gather lived experience data.

Photovoice (in WP1) 'gives the lens' to participants to take photographs and caption them to share their experiences [28]. It is a creative methodology that enables people to express themselves and initiate critical dialogue about community issues, ultimately influencing policy [29]. Research has shown that photovoice has positive effects on participants, leading to feelings of empowerment and social awareness about their environment. Through photovoice, participants can portray experiences that cannot be expressed with words alone, and this often leads to innovative solutions that might not have been recognised otherwise. By sharing photo stories, decision makers are sensitised to address complex health and social issues by taking action [30]. Photovoice is particularly effective in engaging groups that are often underrepresented

For peer review only - http://bmjopen.bmj.com/site/about/guidelines.xhtml

in research, such as people with intellectual disabilities, homelessness, indigenous communities, refugees and those in mental health settings [31-33].

WP2 involves conducting biographical narrative interviews (BNI) with a subset of participants from WP1. The BNI methodology assumes that narrative expression can reveal both conscious concerns and unconscious individual, societal, and cultural influences. It explores both the external and internal worlds with a focus on the life story, while also considering the unique historical and societal context of each case [34]. This approach illustrates how personal and social meanings shape actions in everyday life. Additionally, oral histories through personal accounts offer valuable insights into the past and provide a voice to lived experiences [35]. Biographical interviewing is an effective framework for 'unpacking and deconstructing the past' [36]. BNI is typically a multi-session interview process with minimal interviewer intervention.

The information gathered from the photovoice images, their captions (WP1), and BNI related narratives (WP2) will feed into the experience-based codesign (EBCD) (WP3). This is a crucial opportunity for all involved to listen to each other's perspectives and propose solutions, resulting in a workable set of options.

WP4 consists of two phases. Phase 1 is Baseline E Survey to assess the landscape with regard to service provision and how this may change over the course of the research . Phase 2 consists of the NOMAD survey and a semi structured interview to assess the feasibility of implementing proposed co-designed solutions or models and identify potential barriers to their broader implementation.

For peer review only - http://bmjopen.bmj.com/site/about/guidelines.xhtml

Setting

This is a multicentre study and will be conducted in seven local systems and three participant identification centres (PIC) situated in London, Birmingham (with a PIC site in Worcestershire), Oxford (with a PIC site in Oxfordshire), Derby, Leicester, Braford (with a PIC site in Lancashire), and Northumberland. The chosen recruitment sites are diverse in terms of ethnic populations and geographic locations, encompassing both urban and rural areas across England.

Participants

Throughout the study, it will be crucial to genuinely highlight the voices of marginalised populations. To achieve this, we will involve a patient and public involvement (PPI) group in all stages of this research. We will recruit 80 patients for WP1, comprised of 20 from each urban area and their PIC sites as well as 10 patients from rural places and their PIC sites. We will over-recruit to account for 25% attrition. Patients are welcome to bring in carers or friends to offer them support or act as their advocate. We will collaborate with CRNs and NHS Trusts to reach out to potential participants through local services, such as primary and secondary care, and social care. We will also work with NGOs, local faith communities, and networks of practice and research for recruitment. We will use purposive sampling to ensure that a diverse range of experiences are captured in respect of age, ethnicity, levels of deprivation, gender and religion [37].

We will offer support to ensure that non-English speaking people can participate. Our team will attend board meetings, working groups, and distribute information on staff intranets and throughout the local trust to raise awareness about the research. We will recruit 20 carers and 20 HCP for WP1 (PV) nationally. Recruitment for healthcare professionals and carers will follow the same process.

For peer review only - http://bmjopen.bmj.com/site/about/guidelines.xhtml

For WP2, we will recruit 20 patients who took part in WP1. For WP3 and 4, we will work with local partners to identify key stakeholders. A small number of diverse service providers, patients, and carers will be recruited for EBCD (WP3). For readiness for implementation and feasibility evaluation (WP4.1), we will conduct a survey with 120 participants. This will be followed by a process evaluation (WP4.2) consisting of a NOMAD survey (n=120) and interviews (n=45) participants, including patient participants, informal carers, and healthcare professionals.

Patient and public involvement

Patient and public involvement (PPI) is an essential component of good research practice that leads to more relevant and better-designed research, ensures engagement of service users, and results in better outcomes [38]. This study will employ three people with experience of mental health issues as researchers to work as part of the research team, recruiting participants, co-facilitating workshops, analysing data and sharing findings.

The entire research will be supported by a six-person Lived Experience Advisory Panel (LEAP), ensuring experiential expertise can inform the work at each stage including design, conduct, and dissemination. The LEAP will be hired by McPin, a voluntary organisation that hires people with experience of mental health issues on research studies. The LEAP will meet approximately every 3 months according to the needs and progress of the study. We will design and pilot the information sheets, consent forms, and data collection tools with the LEAP team.

To ensure meaningful public engagement, a person with experience of psychosis and multimorbidities is a co-investigator on the funding application, leads the LEAP team, is a member of the research team, and is employed by the University of Oxford.

For peer review only - http://bmjopen.bmj.com/site/about/guidelines.xhtml

WP1: Photovoice procedure

In each of the local systems, 3 consecutive photovoice workshops will be held in a carefully selected, creative setting that fosters conversation away from clinical environments [28]. If needed, online workshops will be available. In the first workshop, participants will be introduced to the concept of photovoice and how they can use photos and narratives to share their experiences of living with MM (psychosis and two or more long-term conditions), the complexities of care, their experiences with care, and recommendations [28].

Participants will be provided disposable cameras and notebooks during the workshop. They will be asked to return the cameras within two weeks of the workshop, by meeting a member of the local study team who will post the cameras to the central study team. The notebooks will allow for participants to write notes about why they took a particular photo. If they prefer, they can also use their own phones to take photos and send them to the project email address. Participants are also welcome to use their own existing photographs that reflect their care experiences. The project team will consider other creative materials as well, such as drawings, poetry, music and videos.

In the second workshop, participants will be presented with their images and will be guided through a reflective process (prompts presented in Box 1) to generate narratives and captions for a minimum of 3-5 selected images (resulting in a total of 180-300 items). The second workshop will be a drop-in session lasting at least two hours, and can be attended at any time within a 6-8 hour time window. The third workshop will take place a week after the second workshop, and will provide an opportunity for participants to share their images and experiences with each other [39]. The third workshop will be audio recorded and transcribed verbatim. There will be no topic guide, but the conversation will be guided by which images participants choose

For peer review only - http://bmjopen.bmj.com/site/about/guidelines.xhtml

to share, and guided by two overarching questions “what could be different?” and “what made a difference?”.

We will also conduct three PV workshops for 20 carers of those with psychosis and multimorbidities (generating 60-100 images and narrative captions). We will invite twenty professionals responsible for providing care for individuals with psychosis and MM including commissioners, GPs, Physicians, Psychiatrists, Psychologists, Social workers, and Nurses. Selection criteria for PV workshops is presented in Table 1. The professionals will be invited to attend two workshops - an introductory one and a reflective one. We anticipate that both carers and professionals will be from the same regions as the individuals living with psychosis and MM.

Box 1: Prompt questions used to elicit participant experiences

Table 1: Photovoice workshops selection criteria

WP2: Biographic Narrative Interviews Procedure

The interviews, ideally, will be conducted in person, with much flexibility around duration and ways in which people can respond (written, verbal, recorded, etc) [34].

These interviews can last up to a maximum of six hours. However, they don't need to be done in one sitting, rather on separate days, or in short bursts of 20 minutes or an hour, by written or oral responses to prompt questions, or a combination of the above; the exact approach will be personalised, based on individual preferences. Since the interview is about the participant's story-telling, it's crucial not to rush them but rather enable their stories to unfold in the way they would like to tell them [35]. Notably, these interviews are not structured, interrogative, inflexible interviews, but the participant is in charge of their story and the way it is told; therefore, these approaches are enjoyed, and do not place the same burden on participants as surveys.

For peer review only - http://bmjopen.bmj.com/site/about/guidelines.xhtml

Both peer researchers and research assistants will conduct and analyse interviews. We will ensure that adequate training and support in biographical narrative interviewing is undertaken. We will co-produce a distress protocol during the training to ensure that the relevant processes are in place, and that those conducting the interviews are confident in implementing this protocol.

WP3: Co-Design Procedure

The study will follow the Design Council's Double Diamond approach, which involves four stages: discover, define, develop, and deliver [40]. This process helps to gain a better understanding of the issue at hand and take focused action. The aim of these workshops will be to co-design resources and consider their acceptability, feasibility, barriers to implementation, and mechanisms of effect to improve care experiences of people living with psychosis and MM.

- Discover

This phase allows a comprehensive understanding of the issue by engaging with those directly impacted by it. This will be undertaken in WP 1 (PV) and 2 (BNI). The outputs from WP 1 and 2 will be analysed and curated to be presented to the core group in a series of priority setting meetings (3-6 of these) to identify key ‘touch points’ that characterise positive and negative experiences.

- Define and Develop

The information gathered in the Discover phase will be used to define the issue. A range of people will be consulted for their views about the problem and solutions will be co-designed. To establish trust, the same key participants - those who have lived experience of the complex phenomenon being examined, as well as those involved in

For peer review only - http://bmjopen.bmj.com/site/about/guidelines.xhtml

priority setting - will be involved in these phases. The process will entail eight meetings, each lasting two hours.

There will be a series of group meetings, both in-person and online. The initial meetings will involve sharing preliminary ideas and experiences among the core members, followed by identifying patterns and priorities. Once the core group has established their objectives and priorities, they will present these to the wider group for final problem-solving and action-planning. This approach aligns with the psychosocial process employed in Experience-Based Co-Design, which fosters a sense of psychological safety within a group who share similar perspectives, when people are first expressing ideas and exploring how they feel about them.

- Deliver

The resources co-designed in the design and develop phases will be implemented and improved to adjust to real world situations. This phase will be located in work package 4 as part of the acceptability and feasibility work and a process evaluation of resources within sites. Selection criteria for co-design workshops is presented in Table 2.

Table 2: Experience based co-design workshops selection criteria

WP4: Evaluation of readiness for Implementation, Acceptability and Feasibility

Phase 1: Baseline ESurvey:

This will be conducted at T1 (before WP1), T2 (after WP2) and T3 (after WP3). At the beginning of the research (T1) we will survey each of the participating regions, including key decision making stakeholders across all sectors in an ICS to establish: plans for integration of care and services; existing work or planned work on psychosis

For peer review only - http://bmjopen.bmj.com/site/about/guidelines.xhtml

and MM; the priority given to psychosis and multimorbidity, and the relevance of ethnicity in their work; plans over the coming 1,3,5 years; networks of importance and key leaders in the localities that we should contact; invitation to participate in an ongoing network including dissemination of the research and innovations and sharing best practice. The survey will be short, 5-10 questions with likert scales and open text boxes. At T2 and T3 the survey will be repeated. The objective is to look at the broad brushstrokes in relation to any shifts in priorities and to sensitise the research team towards the process evaluation.

We will survey up to 120 ICS leaders and managers and practitioners in participating localities as possible, setting a minimum of 10 and up to 30 key stakeholders identified with our co-investigators and project partners from each locality. We will repeat this after WP2, and after WP3, to evaluate receptivity to implement innovations, any changes in the action plans on psychosis and MM in ethnically diverse samples. We anticipate participatory work itself has an impact, so we will be able to gauge to what extent this happens, and variations over time in a descriptive way to inform an assessment of readiness to change and barriers and facilitators. Participants may be invited back for the process evaluation in the last six months of the project after sharing emergent findings.

Phase 2: Process evaluation

A process evaluation will be conducted using the Normalisation Process Theory (NPT) [41]. NPT is a conceptual framework based on four generative mechanisms that underpin the implementation of complex interventions: coherence (what is the work to be done?); cognitive participation (participants have to buy-in to the work); collective action (what work has to be done to enact and enable new practices?); and reflexive monitoring (what work can be done to help appraise new practices?). We will use in-

For peer review only - http://bmjopen.bmj.com/site/about/guidelines.xhtml

depth semi-structured qualitative interviews with 20-25 professional participants, including decision-makers, commissioners, and health professionals who have used the co-designed resources in hospital, primary, and community care settings. We will also interview up to 20-25 patients and informal carers. Selection criteria for process evaluation is presented in Table 3. Patients and carers will be interviewed separately by default, but may choose to be interviewed together. In such cases, interviews will be conducted in a dyadic fashion. Patient participants will be purposively sampled for maximum variation in relation to age, recruitment site, gender, socio-economic background, and mental health status. Furthermore, Health and social care professionals will be surveyed (n=120) using the NoMAD instrument to gather data on implementation practices and structures [42]. The survey questions will be tailored to identify the potential impact of the co-designed resources and whether they will be integrated into routine usage. This will help in determining the usefulness of the resources and inform their future use.

Table 3: Process evaluation survey and interviews' selection criteria about here

WP5: Economic evaluation

Data collected from WP1-3 will be used for economic evaluation. We will ask patient participants to provide information about their experiences of healthcare services before and after their diagnosis of psychosis. In WP1 during the second workshop we will ask participants to provide a 'road map' or 'life-line' to help them recall events. We will use the theoretical model established by Andersen and Newman to investigate the experience of the care system among patient participants [43]. This model identifies three key types of factors that could influence the use of healthcare services: predisposing features (e.g. age, sex, ethnicity, previous illnesses), enabling features

For peer review only - http://bmjopen.bmj.com/site/about/guidelines.xhtml

(e.g. income, access to care, employment), and needs (e.g. symptoms, diagnosis, functioning) (17). Furthermore, participants will be encouraged to indicate any care they would have preferred to receive but did not, as well as suggest reasons or barriers for why this was the case. The removal of these barriers may have cost implications and could also impact outcomes, which we will explore using economic modelling methods.

Analysis

Data across work packages will be collected in the form of photographic images and captions, written or other reflective accounts of experience, such as drawings and poetry, biographical narratives and group discussions, surveys. Data will also be collected via qualitative interviews and a baseline survey concerning available services and the NoMAD survey for a process evaluation The Client Service Receipt Inventory (CSRI) will be used to record the use of health and social care services.

WP1 Photovoice will be analysed using a combination of framework analysis [44] and polytextual thematic analysis [45, 46] to both organise and understand the data. The work package will render three levels of data: demographic data, images and captions and transcripts from the discussion of the images amongst participants from the third workshop. The following eight steps of polytextual thematic analysis will be used to analyse the data [46] : Step 1 – Understanding the sample, step 2 – Organising the data, step 3 – Individual case analysis, step 4 – Exploring interactions, step 5- combining the individual and group analyses, step 6 – Theme generation, Step 7 – Polytextual thematic analysis [46].

WP2 Biographical Narrative Interviews will be audio recorded and transcribed verbatim. The transcripts will be imported to MAXQDA. Thematic Analysis [47] will be used to code the data, and from this, themes will be generated. The resulting themes

For peer review only - http://bmjopen.bmj.com/site/about/guidelines.xhtml

will be considered alongside the analysis from WP1 to look for points of both similarity and contention. Peer researchers will be supported to also code transcripts and will be part of the team discussions to consider the iteration of higher order themes.

WP3 Experience-Based Co-Design workshops will be audio recorded and transcribed verbatim. The purpose is to identify the context and reasons for decisions about co-design, to address indicated and agreed touchpoints. There may also be realisations that some groups are not going to be well served if a particular design or set of priorities are proposed. The narrative accounts will be described to set out such decisions, rather than needing a formal qualitative analysis process. We will ensure all respective views and positions are represented and the narrowing of options towards consensus is evident. Those participating in the co-design workshops will be given an opportunity to review the transcripts and summary documents.

WP4 is a process evaluation. which has two phases. Phase 1 is a baseline e survey that will take place at T1 (prior to WP1) T2 (after , WP2)) and T3 (after WP3) and will be analysed descriptively using means, standard deviations and 95% confidence intervals. Inductive content analysis will be used for categorising the free text responses. Responses will be descriptively coded and reviewed to identify representative phrases that are characteristic of or overlap with professional groupings.

Phase 2 of WP4 takes place after WP3 and data will be collected via in depth semi-structured interviews and the NoMAD survey [48]. All interview data will be transcribed verbatim and analysed thematically. An initial thematic coding framework will be developed and checked. Codes in each interview will be examined across individual transcripts as well as the entire data set and allocated to the framework. Using aspects

For peer review only

of the constant comparative analysis method broader categories using linked codes will be developed across interviews. Analysis will be guided by items of NPT to structure patients', carers'; and professionals' views about acceptability and implementation readiness of the intervention.

The NoMAD survey tool includes 20 items that reflect the four core NPT constructs and three items to capture data about participants' views about the likelihood that an intervention can become a routine part of their work. The 20-items that compare responses across the four NPT constructs are scored using a 5-point Likert scale to indicate the level of agreement, where 1= strongly agree, 3=neutral and 5=strongly disagree. The additional three generic questions about suitability of the intervention for routine practice are scored using a response scale from zero to 10, with 0-not at all; 5= somewhat; and 10 =completely. We will analyse NoMAD responses descriptively, reporting absolute and relative frequencies, along with means and standard deviations and 95% confidence intervals. Where items are negatively phrased responses will be inversely scored. Total scores will not be calculated.

WP5 Health Economics is using The Client Service Receipt Inventory (CRSI) to record the use of health and social care services. These data will be combined with appropriate unit cost information to calculate care costs. Regression models will be used to identify significant demographic and clinical predictors of cost. The potential cost of services not received but perceived as important by participants will also be calculated. Decision modelling will examine the impact of removing care barriers on costs.

For peer review only - http://bmjopen.bmj.com/site/about/guidelines.xhtml

Ethics and Dissemination

All participants will be provided with detailed information about what participation entails through a participant information sheet, as well as through verbal explanation to ensure full understanding [49]. Only individuals with the capacity to consent will be included in the study, and informed consent will be obtained at each phase of the research. Participants will be informed that they are free to leave the study at any time without having to provide any reasons. Participants will be assured that they will not face any negative consequences regarding their care. Given that individuals with MM and psychosis from marginalised communities are often more vulnerable, the research team and local systems will take measures to protect, empower, and provide necessary support to participants throughout the research process [16]. This will prevent any potential power imbalances and foster a relationship of trust between researchers and participants. Furthermore, the research methodologies employed seek to minimise stress, provide opportunities for participants to express their artistic abilities, and do not necessitate the verbalisation of emotionally challenging experiences [50]. The confidentiality and anonymity of participants' personal information shall be strictly maintained. The photos and captions will not reveal the identities of the participants unless they make a request for disclosure.

This project will prioritise knowledge exchange and collaborative working. We will take care to disseminate the outputs so the participants do not feel disempowered or lose ownership. To create awareness, we will use online resources such as social media platforms like Twitter, Instagram, and TikTok, as well as a dedicated website and monthly newsletters. Photos and captions taken with WP1 will be shared through exhibitions across local sites to showcase the experiences of individuals with psychosis and MM. The hope is that these shared experiences will inspire service

For peer review only - http://bmjopen.bmj.com/site/about/guidelines.xhtml

providers and policy makers to work towards improving services. Furthermore, academic publications will be authored along with partners to further the discussions about using creative methodologies and reducing disparities among diverse groups.

Author contributions

KB is the PI and led the writing of the grant and intellectual work, and prepared revisions, taking feedback from all co-investigators around their specific methodological expertise; UZ, TM, NK contributed to revisions of the protocol before (UZ) and after (TM, NK) securing funding. TM and DJ bring lived experience expertise to design and delivery. Co-investigators' contribute additional methodological expertise: PM-economics; ML-Experience based co-design; DE. PC-evaluation and implementation; JA, FK-sociology and inequalities and qualitative research; FK, RU, RM, KB- photovoice; GH, RU, SG-adversity and life-course epidemiology. RM is a co-investigator who developed the initial protocol for ethics from the funding application.

Funding statement

This study was funded by NIHR HSDR panel (funding reference, NIHR 151887). KB and RM are part supported by Oxford Health NIHR – BRC, and Oxford-TV NIHR-ARC.

Conflicts of interest

None

For peer review only

References

1. Halvorsrud, K., et al., Identifying evidence of effectiveness in the co-creation of research: a systematic review and meta-analysis of the international healthcare literature. Journal of Public Health (Oxford, England), 2021. 43(1): p. 197-208.
2. Das-Munshi, J.D., et al., Ethnicity and excess mortality in severe mental illness: a cohort study. The Lancet. Psychiatry, 2017. 4(5): p. 389-399.
3. Das-Munshi, J., et al., Type 2 diabetes mellitus in people with severe mental illness: inequalities by ethnicity and age. *Cross-sectional* analysis of 588 408 records from the UK. Diabetic medicine, 2017. 34(7): p. 916-924.
4. Hassan, S., et al., A qualitative study exploring the barriers and facilitators of implementing a cardiovascular disease risk reducing intervention for people with severe mental illness into primary care contexts across England: the 'PRIMROSE' trial. BMC Health Services Research, 2020. 20(1).
5. Excellence, N.I.f.H.a.C. Multimorbidity: clinical assessment and management. NICE Guidelines 2016 23rd October 2023]; Available from: https://www.nice.org.uk/guidance/ng56.
6. Owen, R.K., et al., Effect on life expectancy of temporal sequence in a multimorbidity cluster of psychosis, diabetes, and congestive heart failure among 1·7 million individuals in Wales with 20-year follow-up: a retrospective cohort study using linked data. The Lancet. Public health, 2023. 8(7): p. e535-e545.
7. Catalao, R., et al., Ethnic inequalities in mental and physical multimorbidity in women of reproductive age: a data linkage cohort study. BMJ open, 2022. 12(7).
8. Fonseca de Freitas, D., et al., Ethnic inequities in multimorbidity among people with psychosis: a retrospective cohort study. Epidemiology and psychiatric sciences, 2022. 31: p. e52-e52.
9. England, N. Improving physical Healthcare for people living with severe mental illness in primary care: Guidance for CCG's. 2018 23rd October 2023]; Available from: https://www.england.nhs.uk/publication/improving-physical-healthcare-for-people-living-with-severe-mental-illness-smi-in-primary-care-guidance-for-ccgs/.
10. Zahid, U., et al., Ethnic inequality, multimorbidity and psychosis: can a syndemic framework resolve disputed evidence? NPJ schizophrenia, 2023. 9(1): p. 37-37.
11. Singer, M.P., et al., Syndemics and the biosocial conception of health. The Lancet (British edition), 2017. 389(10072): p. 941-950.
12. Bhui, B.K., Impulsivity, Attachment, and Relational Psychopathology. British journal of psychiatry, 2018. 213(3): p. 570-570.
13. Coid, J., et al., Ethnic disparities in psychotic experiences explained by area-level syndemic effects. The British Journal of Psychiatry, 2020. 217: p. 555-561.
14. Hackett, R.A., et al., Racial discrimination and health: a prospective study of ethnic minorities in the United Kingdom. BMC public health, 2020. 20(1): p. 1652-1652.
15. Pefoyo, A.J., et al., The increasing burden and complexity of multimorbidity. BMC Public Health, 2015. 15: p. 415.
16. Willadsen, T.G., et al., Symptom burden in multimorbidity: a population-based combined questionnaire and registry study from Denmark. BMJ Open, 2021. 11(4): p. e041877.
17. Hounkpatin, H.O., et al., Change in treatment burden among people with multimorbidity: a follow-up survey. British Journal of General Practice, 2022.
18. Rodgers, M., et al., Integrated care to address the physical health needs of people with severe mental illness: A mapping review of the recent evidence on barriers, facilitators and evaluations. International journal of integrated care, 2018. 18(1): p. 9-9.
19. Whitty, C.J.M. and F.M. Watt, Map clusters of diseases to tackle multimorbidity. Nature (London), 2020. 579(7800): p. 494-496.

For peer review only - http://bmjopen.bmj.com/site/about/guidelines.xhtml

20. Langan, J., S.W. Mercer, and D.J. Smith, Multimorbidity and mental health: can psychiatry rise to the challenge? British journal of psychiatry, 2013. 202(6): p. 391-393.
21. Parker, S.G., et al., Priorities for research in multiple conditions in later life (multi-morbidity): findings from a James Lind Alliance Priority Setting Partnership. Age and ageing, 2019. 48(3): p. 401-406.
22. Wang, C. and M.A. Burris, Photovoice: Concept, Methodology, and Use for Participatory Needs Assessment. Health Education & Behavior, 1997. 24(3): p. 369-387.
23. Council, T.D. The Double Diamond. 2023; Available from: https://www.designcouncil.org.uk/our-resources/framework-for-innovation/.
24. Chen, Y.H., M. Karimi, and M. Rutten-van Molken, The disease burden of multimorbidity and its interaction with educational level. PLoS One, 2020. 15(12): p. e0243275.
25. Egid, B.R., et al., 'You want to deal with power while riding on power': global perspectives on power in participatory health research and co-production approaches. Bmj Global Health, 2021. 6(11).
26. Bailey, J., et al., Getting at Equality: Research Methods Informed by the Lessons of Intersectionality. International Journal of Qualitative Methods, 2019. 18.
27. Moll, S., et al., Are you really doing 'codesign'? Critical reflections when working with vulnerable populations. Bmj Open, 2020. 10(11).
28. Sutton-Brown, C.A., Photovoice: A Methodological Guide. Photography and Culture, 2014. 7(2): p. 169-185.
29. Budig, K., et al., Photovoice and empowerment: evaluating the transformative potential of a participatory action research project. Bmc Public Health, 2018. 18.
30. Nykiforuk, C.I.J., H. Vallianatos, and L.M. Nieuwendyk, Photovoice as a Method for Revealing Community Perceptions of the Built and Social Environment. International Journal of Qualitative Methods, 2011. 10(2): p. 103-124.
31. Jurkowski, J.M., Photovoice as participatory action research tool for engaging people with intellectual disabilities in research and program development. Intellectual and Developmental Disabilities, 2008. 46(1): p. 1-11.
32. Campbell, R.B., et al., The challenges of managing diabetes while homeless: a qualitative study using photovoice methodology. Canadian Medical Association Journal, 2021. 193(27): p. E1034-E1041.
33. Bennett, B., et al., The Gaataa'aabing Visual Research Method: A Culturally Safe Anishinaabek Transformation of Photovoice. International Journal of Qualitative Methods, 2019. 18.
34. Wengraf, T., Qualitative Research Interviewing. First ed. 2001: SAGE Publications Ltd.
35. Chamberlayne P, B.J., Wengraf T, The Turn to Biographical Methods in Social Science: Comparative Issues and Examples.: London: Routledge.
36. Brooks, E. and R. Dallos, Exploring young women's understandings of the development of difficulties: a narrative biographical analysis. Clin Child Psychol Psychiatry, 2009. 14(1): p. 101-15.
37. Palinkas, L.A., et al., Purposeful Sampling for Qualitative Data Collection and Analysis in Mixed Method Implementation Research. Adm Policy Ment Health, 2015. 42(5): p. 533-44.
38. Brett, J., et al., A systematic review of the impact of patient and public involvement on service users, researchers and communities. Patient, 2014. 7(4): p. 387-95.
39. Mooney, R., et al., Experience-based investigation and codesign of approaches to prevent and reduce Mental Health Act use (CO-PACT): a protocol. BMJ Open, 2022. 12(2): p. e060086.
40. Council, D. Framework for Innovation 2005 26/10/2023]; Available from: https://www.designcouncil.org.uk/our-resources/framework-for-innovation/#:~:text=Design%20Council's%20Double%20Diamond%20clearly,focused%20action%20(convergent%20thinking). .

For peer review only - http://bmjopen.bmj.com/site/about/guidelines.xhtml

41. Murray, E., et al., Normalisation process theory: a framework for developing, evaluating and implementing complex interventions. Bmc Medicine, 2010. 8.
42. Finch, T.L., et al., Improving the normalization of complex interventions: part 2-validation of the NoMAD instrument for assessing implementation work based on normalization process theory (NPT). Bmc Medical Research Methodology, 2018. 18.
43. Babitsch, B., D. Gohl, and T. von Lengerke, Re-revisiting Andersen's Behavioral Model of Health Services Use: a systematic review of studies from 1998-2011. Psychosoc Med, 2012. 9: p. Doc11.
44. Spencer, L.R., J., Lewis, J. & Dillon, L., Quality in Qualitative Evaluation: A framework for assessing research evidence, G.C.S.R.s. Office, Editor. 2003, Crown Copyright.
45. Gleeson, K., Polytextual thematic analysis for visual data: analysing visual images, in A Handbook of Visual Methods in Psychology, P. Reavey, Editor. 2021, Routledge: Abingdon. p. 536-554.
46. Mooney, R., et al., Analysing multimodal data that have been collected using photovoice as a research method. BMJ open, 2023. 13(4): p. e068289-e068289.
47. Braun, V. and V. Clarke, Reflecting on reflexive thematic analysis. Qualitative research in sport, exercise and health, 2019. 11(4): p. 589-597.
48. May, C., Rapley, T., Mair ,F.S., Treweek, S., Murray, E., Balllini, L., Macfarlane, A. Girling, M. and Finch, T.L. . Normalization Process Theory On-line Users' Manual, Toolkit and NoMAD instrument. 2015 23rd October 2023]; Available from: https://normalization-process-theory.northumbria.ac.uk/.
49. Lieberman, J.A., et al., Ethical principles and practices for research involving human participants with mental illness. Psychiatric Services, 2006. 57(4): p. 552-557.
50. Catalani, C. and M. Minkler, Photovoice: a review of the literature in health and public health. Health Educ Behav, 2010. 37(3): p. 424-51.

For peer review only

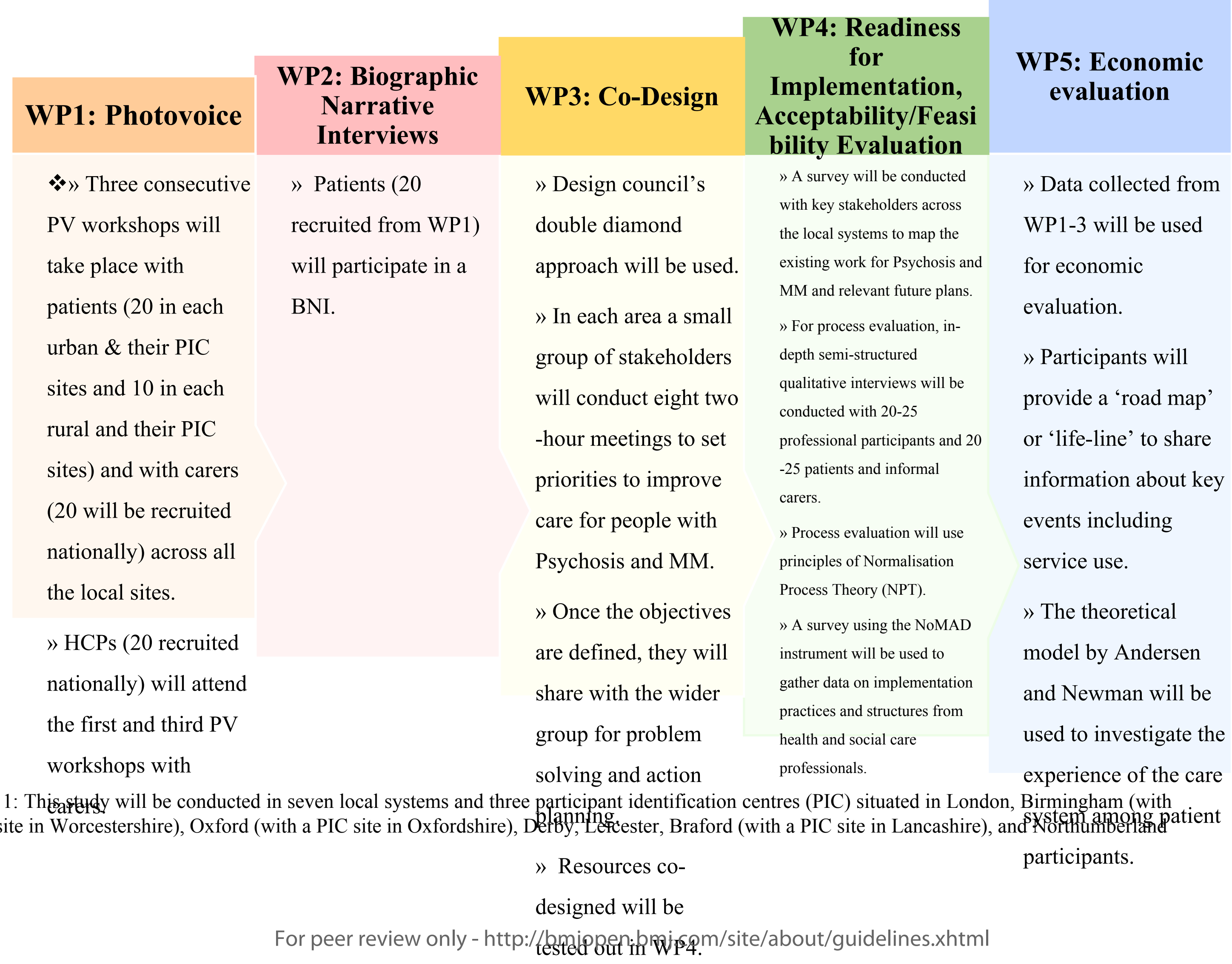

Figure 1: This study will be conducted in seven local systems and three participant identification centres (PIC) situated in London, Birmingham (with a PIC site in Worcestershire), Oxford (with a PIC site in Oxfordshire), Derby, Leicester, Braford (with a PIC site in Lancashire), and Northumberland .

Box 1:

Prompt questions used to elicit participant experiences

- Describe what you see in this photo and how it makes you feel?
- Why did you take a photo of this?
- What does this photo tell us about you?
- How can it provide opportunities to improve understanding about your illness and life experiences?
- Do you have suggestions for improving your care?
- In your care experiences, what helped and what did not work?
- How can inequalities arise and be reduced?

For peer review only

Table 1: Photovoice workshops selection criteria

Inclusion	Exclusion
**For Patient Participants**	
• Over the age of 18.	• Acute distress or immediate health problems that must be prioritised (e.g. suicidal thinking/intent, urgent need for medical intervention or series of treatments).
• Index diagnosis of psychosis and at least two of the following conditions known to lead to premature death; diabetes, cardio-respiratory, kidney and liver disease.	People with only psychosis and a common mental disorder, such as anxiety or mood disorders.
• We will seek to ensure representation of those facing most inequality; for example, Black Caribbean and Black African, as well as Indian, Pakistani and Bangladeshi groups of men and women; and mixed groups, Eastern Europeans, Asylum Seekers/Refugees, and White British identifying people.	
• Willing and able to provide consent.	

For peer review only - http://bmjopen.bmj.com/site/about/guidelines.xhtml

**For Carers**	
• Over the age of 18.	• Formal carers.
• Informal caring responsibilities for someone who meets the patient selection criteria.	• Unable to provide consent.
**For Healthcare Professionals**	
• Over the age of 18.	• Less than 6 months experience of working with people with Psychosis.
• Working in any capacity in any of the systems that work with people with Psychosis (such as social work, psychiatry, psychology, general practice and nursing).	• Unable to provide consent.

For peer review only

Table 2: Experience based co-design workshops selection criteria

Inclusion	Exclusion
• People with lived experience of receiving care or delivering care in the relevant systems, and people with the power to make decisions and implement changes in the system.	• No lived experience of psychosis or MM or working with people with psychosis or MM.
• Willing and able to provide consent.	

Table 3: Process evaluation survey and interviews' selection criteria

Inclusion	Exclusion
**For survey**	
• Participants will be healthcare professionals working in primary care practices, secondary care mental health	• Those in non-professional or non-NHS facing job roles will be excluded from the survey.

services, and/or employees of Integrated Care Systems in clinical and non-clinical roles.	
• Willing and able to provide consent.	
**For interviews**	
• Eligibility criteria for patient participants will be the same as WP1.	• Exclusion criteria for patient participants will be the same as WP1.
• Eligibility criteria for carers will be the same as WP1.	• Exclusion criteria for patient participants will be the same as WP1.
• Health care professionals will be eligible if they are employed in primary care or secondary care mental health services in the same geographic footprint of participating ICSs.	• Healthcare professionals working outside the participating ICS case study regions will be excluded.

For peer review only

BMJ Open

Experience-based Investigation and Co-design of Psychosis Centred Integrated Care Services for Ethnically Diverse People with Multimorbidity (CoPICS): Study Protocol.

Journal:	*BMJ Open*
Manuscript ID	bmjopen-2024-084121.R1
Article Type:	Protocol
Date Submitted by the Author:	16-Jan-2024

Complete List of Authors:	Bhui , Kamaldeep; University of Oxford, Psychiatry ; Queen Mary University of London<br>Joseph , Doreen ; University of Oxford, Psychiatry<br>Khan, Nimra; University of Oxford, Psychiatry<br>Morrey, Tara; University of Oxford, Psychiatry<br>Mooney, Roisin; University of Oxford, Psychiatry<br>Zahid, Uzma; KCL<br>Mackay, Tanya; McPin Foundation<br>Larkin, Michael; Aston University<br>Keating, Frank; Royal Holloway University of London, Department of Social Work<br>McCrone, Paul; University of Greenwich<br>Upthegrove, Rachel; University of Birmingham, Department of Psychiatry, School of Psychology and College of Medical and Dental Sciences; Forward Thinking Birmingham, Early Intervention Service<br>Griffiths, Sian Lowri; University of Birmingham<br>Edge, Dawn; Univ Manchester<br>Coventry, Peter; University of York,<br>Arday, Jason; Cambridge University<br>Hosang, Georgina M.; Queen Mary University of London

**Primary Subject Heading**:	Mental health
Secondary Subject Heading:	Health services research, Health policy, Public health, Qualitative research
Keywords:	Schizophrenia & psychotic disorders < PSYCHIATRY, Chronic Disease, Health Services

SCHOLARONE™
Manuscripts

Word count:

Main text 4693

Abstract 231

Experience-based Investigation and Co-design of Psychosis Centred Integrated Care Services for Ethnically Diverse People with Multimorbidity (CoPICS): Study Protocol.

*Corresponding author Prof. Kamaldeep Bhui, Dept of Psychiatry, Warneford Hospital, Oxford OX3 7JX. Kam.bhui@psych.ox.ac.uk

University of Oxford
*Kamaldeep Bhui, professor
Doreen Joseph, lived experience expert
Nimra Khan, post-doctoral researcher
Tara Morrey, post-doctoral researcher
Roisin Mooney, post-doctoral researcher

King's College London
Uzma Zahid, Research Fellow

The McPin Foundation
Tanya Mackay, lived experience lead

Aston University, Birmingham
Michael Larkin, professor

Royal Holloway University of London
Frank Keating, professor

Greenwich University
Paul McCrone, professor

University of Birmingham
Rachel Upthegrove, professor
Sian Griffiths, research fellow

University of Manchester
Dawn Edge, professor

University of York
Peter Coventry, professor

University of Cambridge
Jason Arday, professor

Queen Mary University of London,
Georgina Hosang, Reader

For peer review only

Abstract

Introduction: Ethnic minorities (also called racialised groups), are more likely to experience severe mental illness (SMI). People with SMI are more likely to experience multimorbidity (MM), making psychosis amongst racialised groups more likely to lead to MM, poor outcomes, disability, and premature mortality.

Methods and analysis: This NIHR funded study [151887] seeks to use innovative participatory methods including photovoice and biographical narrative interviews in urban and rural areas of England to assemble experience data. This data will be subjected to polytextual thematic analysis, and alongside pictures and captions, will inform an experienced based co-design of interventions, the implementation of which will be evaluated. There will be an economic analysis and a process evaluation of the implementation.

Ethics and dissemination: This programme of work has received ethical (IRAS 322421; Newcastle North Tyneside Research Ethics Committee 23/NE/0143) and sponsor approval. The findings will be disseminated in galleries showing the creative work, as lay and academic summaries and infographics; as practice briefings for practitioners, commissioners, and policy makers; peer reviewed publications.

Strengths and limitations:

- The study design was informed by lived experience involvement, as will be the delivery and dissemination of findings.
- To recruit groups usually under-represented in research, we use creative, empowering, and engaging participatory and biographical narrative methods.
- We will recruit ethnically diverse adults with psychosis and two or more other long term conditions, from NHS, community, rural and urban sites.

For peer review only

- The study will take place in England.
- Implementation and evaluation of co-designed approaches will be influenced by contextual factors.

For peer review only

For peer review only - http://bmjopen.bmj.com/site/about/guidelines.xhtml

Introduction

Black ethnic groups have a higher risk of developing severe mental illness (SMI) such as schizophrenia, and both affective and non-affective psychoses. This risk is also higher for other diverse ethnic groups [1]. In racialised populations with SMI physical health conditions are also more prevalent, such as asthma, cardiac disease and diabetes in South Asians, and asthma, high blood pressure and stroke in Black groups [1-4]. NICE [5] defines 'multimorbidity' as the presence of two or more long term health conditions one of which is a physical health condition of long duration such as diabetes or infectious disease, and the other may be a further long-term physical condition or SMI. Multimorbidity can substantially reduce life expectancy [4, 6].

Amongst diverse ethnic populations health inequities are more prevalent [7, 8]. SMI also contributes significantly to health inequality gaps in England [2, 9]. One of the main reasons for this, is that physical health needs in people with SMI's are not prioritised [9].

Existing evidence about the prevalence of multimorbidity in racialised populations suggests that complex social determinants independently or in combination raise the risk of multimorbidity [10]. One pertinent lens through which to view these intersecting elements is via the concept of a syndemic [11]. A syndemic refers to a clustering of specific populations and health conditions which interact with contextual and social factors. In racialised populations at a macro level this clustering may include elements such as: constructions of health and health care, structural racism, ethnicity, environment, socio-economic status and poverty [12-14]. At a micro level this may include facets like individual lived experience, childhood adversity, identity, internalised hatred, attachment and esteem [12-14].

For peer review only - http://bmjopen.bmj.com/site/about/guidelines.xhtml

Such complex bio-social pathways amongst racialised populations appear to drive the onset of SMI's such as psychosis and other long-term physical or mental health conditions. People living with psychosis and MM face a huge burden of medical and social problems, leading to poorer health outcomes, high rates of polypharmacy, treatment burden, lower quality of life, feelings of vulnerability, disability, and even premature mortality [15-17]. Due to health-related burdens, it can be challenging for those with MM to participate in traditional forms of research.

Therefore, there is a need for integrated health and social care research and consequent interventions to attend to social determinants and socio-economic status, as these can complicate recovery and lead to inequality [8, 10]. There are issues in delivering integrated care. Whilst there is a need to focus on both the mental and physical health of those with multimorbidity, physical and mental health care is not fully integrated in training professionals or across care services [18-20]. For example, integrated service provision may be hampered by the complexity of care for patients with multimorbidity and issues in communication across physical and mental healthcare services [18-20]. There is some research focus on what could be done in primary care, such as developing staff skills and training for primary care practitioners in cardio-vascular disease prevention amongst those with SMI [4]. In secondary care suggestions for improvement include empowering SMI staff and service users to remove barriers to delivering and accessing integrated care, improved communication amongst healthcare professionals and better information technology to support both communication and information access [18].

Given the higher risk of multimorbidity amongst racialised populations, the effect of multimorbidity on life expectancy, and the priority of delivering integrated care to those with multimorbidity [5] this research aims to:

For peer review only - http://bmjopen.bmj.com/site/about/guidelines.xhtml

- support NICE guidance [5] in improving care for multimorbidity through exploring how current care and interventions are experienced by patients, carers and health care professionals.
- contribute to evolving strategies that prevent the development of multiple conditions
- understand more about causation and the role of ethnicity and health and social care in meeting the needs of people living with multimorbidity, aligning with the James Lind Alliance priorities [21].
- facilitate the development of preventative interventions by ensuring diverse patient experiences are considered in care, especially for socially excluded groups who do not access care or find existing care unhelpful.
- draw upon innovative and inclusive methods such as photovoice [22] and experience-based co-design [23] and recruit groups that are historically under-represented in research.

Methods

Design

This is a mixed methods study investigating the experiences of people living with psychosis and multimorbidities (MM), with emphasis on those who are marginalised such as racialised communities, and service providers, commissioners, informal carers, and other important stakeholders identified across the health system.

Figure 1: Study design

Methodological Innovations

For peer review only

Research studies frequently lack representation from marginalised and racialised communities [24]. Conventional data collection methods can perpetuate power imbalances. There is a need for supportive data collection methods that engage underrepresented groups [25]. Collaborative and participatory research methods offer innovative ways to engage with communities that have been historically marginalised [25]. Participatory research and storytelling techniques will be used to inform an experience-based co-design and process evaluation of implementation [26]. These methods equalise power dynamics between researchers and participants, and create a secure and inclusive environment. In addition, the incorporation of art-based techniques can draw out personal experiences and cultivate potential solutions in a gentle and iterative manner. Thus, co-design approaches can include the use of creative tools including storyboards, videos, photos, clay, and Lego, which have been shown to evoke deeper insights [27]. Therefore, we will employ Photovoice (PV) in work package 1 (WP1) and Biographical Narrative Interviews (BNI) in work package 2 (WP2) to gather lived experience data (see Figure 1).

Photovoice (in WP1) ‘gives the lens’ to participants to take photographs and caption them to share their experiences [28]. It is a creative methodology that enables people to express themselves and initiate critical dialogue about community issues, ultimately influencing policy [29]. Research has shown that photovoice has positive effects on participants, leading to feelings of empowerment and social awareness about their environment. Through photovoice, participants can portray experiences that cannot be expressed with words alone, and this often leads to innovative solutions that might not have been recognised otherwise. By sharing photo stories, decision makers are sensitised to address complex health and social issues by taking action [30]. Photovoice is particularly effective in engaging groups that are often underrepresented

For peer review only - http://bmjopen.bmj.com/site/about/guidelines.xhtml

in research, such as people with intellectual disabilities, homelessness, indigenous communities, refugees and those in mental health settings [31-33].

WP2 involves conducting biographical narrative interviews (BNI) with a subset of participants from WP1. The BNI methodology assumes that narrative expression can reveal both conscious concerns and unconscious individual, societal, and cultural influences. It explores both the external and internal worlds with a focus on the life story, while also considering the unique historical and societal context of each case [34]. This approach illustrates how personal and social meanings shape actions in everyday life. Additionally, oral histories through personal accounts offer valuable insights into the past and provide a voice to lived experiences [35]. Biographical interviewing is an effective framework for 'unpacking and deconstructing the past' [36]. BNI is typically a multi-session interview process with minimal interviewer intervention.

The information gathered from the photovoice images, their captions (WP1), and BNI related narratives (WP2) will feed into the experience-based codesign (EBCD) (WP3). This is a crucial opportunity for all involved to listen to each other's perspectives and propose solutions, resulting in a workable set of options.

WP4 consists of two phases. Phase 1 is Baseline E Survey to assess the landscape with regard to service provision and how this may change over the course of the research . Phase 2 consists of the NOMAD survey and a semi structured interview to assess the feasibility of implementing proposed co-designed solutions or models and identify potential barriers to their broader implementation.

For peer review only - http://bmjopen.bmj.com/site/about/guidelines.xhtml

Setting

This is a multicentre study and will be conducted in seven local systems and three participant identification centres (PIC) situated in London, Birmingham (with a PIC site in Worcestershire), Oxford (with a PIC site in Oxfordshire), Derby, Leicester, Braford (with a PIC site in Lancashire), and Northumberland. The chosen recruitment sites are diverse in terms of ethnic populations and geographic locations, encompassing both urban and rural areas across England.

Participants

Throughout the study, it will be crucial to genuinely highlight the voices of marginalised populations. To achieve this, we will involve a patient and public involvement (PPI) group in all stages of this research. We will recruit 80 patients for WP1, comprised of 20 from each urban area and their PIC sites as well as 10 patients from rural places and their PIC sites. We will over-recruit to account for 25% attrition. Patients are welcome to bring in carers or friends to offer them support or act as their advocate. We will collaborate with CRNs and NHS Trusts to reach out to potential participants through local services, such as primary and secondary care, and social care. We will also work with NGOs, local faith communities, and networks of practice and research for recruitment. We will use purposive sampling to ensure that a diverse range of experiences are captured in respect of age, ethnicity, levels of deprivation, gender and religion [37].

We will offer support to ensure that non-English speaking people can participate. Our team will attend board meetings, working groups, and distribute information on staff intranets and throughout the local trust to raise awareness about the research. We will recruit 20 carers and 20 HCP for WP1 (PV) nationally. Recruitment for healthcare professionals and carers will follow the same process.

For peer review only - http://bmjopen.bmj.com/site/about/guidelines.xhtml

For WP2, we will recruit 20 patients who took part in WP1. For WP3 and 4, we will work with local partners to identify key stakeholders. A small number of diverse service providers, patients, and carers will be recruited for EBCD (WP3). For readiness for implementation and feasibility evaluation (WP4.1), we will conduct a survey with 120 participants. This will be followed by a process evaluation (WP4.2) consisting of a NOMAD survey (n=120) and interviews (n=45) participants, including patient participants, informal carers, and healthcare professionals.

Patient and public involvement

Patient and public involvement (PPI) is an essential component of good research practice that leads to more relevant and better-designed research, ensures engagement of service users, and results in better outcomes [38]. This study will employ three people with experience of mental health issues as researchers to work as part of the research team, recruiting participants, co-facilitating workshops, analysing data and sharing findings.

The entire research will be supported by a six-person Lived Experience Advisory Panel (LEAP), ensuring experiential expertise can inform the work at each stage including design, conduct, and dissemination. The LEAP will be hired by McPin, a voluntary organisation that hires people with experience of mental health issues on research studies. The LEAP will meet approximately every 3 months according to the needs and progress of the study. We will design and pilot the information sheets, consent forms, and data collection tools with the LEAP team.

To ensure meaningful public engagement, a person with experience of psychosis and multimorbidities is a co-investigator on the funding application, leads the LEAP team, is a member of the research team, and is employed by the University of Oxford.

For peer review only - http://bmjopen.bmj.com/site/about/guidelines.xhtml

WP1: Photovoice procedure

In each of the local systems, 3 consecutive photovoice workshops will be held in a carefully selected, creative setting that fosters conversation away from clinical environments [28]. If needed, online workshops will be available. In the first workshop, participants will be introduced to the concept of photovoice and how they can use photos and narratives to share their experiences of living with MM (psychosis and two or more long-term conditions), the complexities of care, their experiences with care, and recommendations [28].

Participants will be provided disposable cameras and notebooks during the workshop. They will be asked to return the cameras within two weeks of the workshop, by meeting a member of the local study team who will post the cameras to the central study team. The notebooks will allow for participants to write notes about why they took a particular photo. If they prefer, they can also use their own phones to take photos and send them to the project email address. Participants are also welcome to use their own existing photographs that reflect their care experiences. The project team will consider other creative materials as well, such as drawings, poetry, music and videos.

In the second workshop, participants will be presented with their images and will be guided through a reflective process (prompts presented in Box 1) to generate narratives and captions for a minimum of 3-5 selected images (resulting in a total of 180-300 items). The second workshop will be a drop-in session lasting at least two hours, and can be attended at any time within a 6-8 hour time window. The third workshop will take place a week after the second workshop, and will provide an opportunity for participants to share their images and experiences with each other [39]. The third workshop will be audio recorded and transcribed verbatim. There will be no topic guide, but the conversation will be guided by which images participants choose

For peer review only - http://bmjopen.bmj.com/site/about/guidelines.xhtml

to share, and guided by two overarching questions "what could be different?" and "what made a difference?".

We will also conduct three PV workshops for 20 carers of those with psychosis and multimorbidities (generating 60-100 images and narrative captions). We will invite twenty professionals responsible for providing care for individuals with psychosis and MM including commissioners, GPs, Physicians, Psychiatrists, Psychologists, Social workers, and Nurses. Selection criteria for PV workshops is presented in Table 1. The professionals will be invited to attend two workshops - an introductory one and a reflective one. We anticipate that both carers and professionals will be from the same regions as the individuals living with psychosis and MM.

Box 1:

Prompt questions used to elicit participant experiences

- Describe what you see in this photo and how it makes you feel?
- Why did you take a photo of this?
- What does this photo tell us about you?
- How can it provide opportunities to improve understanding about your illness and life experiences?
- Do you have suggestions for improving your care?
- In your care experiences, what helped and what did not work?
- How can inequalities arise and be reduced?

Table 1: Photovoice workshops selection criteria

Inclusion	Exclusion
**For Patient Participants**	
• Over the age of 18.	• Acute distress or immediate health problems that must be prioritised (e.g. suicidal thinking/intent, urgent need for

	medical intervention or series of treatments).
• Index diagnosis of psychosis and at least two of the following conditions known to lead to premature death; diabetes, cardio-respiratory, kidney and liver disease.	People with only psychosis and a common mental disorder, such as anxiety or mood disorders.
• We will seek to ensure representation of those facing most inequality; for example, Black Caribbean and Black African, as well as Indian, Pakistani and Bangladeshi groups of men and women; and mixed groups, Eastern Europeans, Asylum Seekers/Refugees, and White British identifying people.	
• Willing and able to provide consent.	
**For Carers**	
• Over the age of 18.	• Formal carers.
• Informal caring responsibilities for someone who meets the patient selection criteria.	• Unable to provide consent.
**For Healthcare Professionals**	
• Over the age of 18.	• Less than 6 months experience of working with people with Psychosis.
• Working in any capacity in any of the systems that work with people with Psychosis (such as social work, psychiatry, psychology, general practice and nursing).	• Unable to provide consent.

For peer review only - http://bmjopen.bmj.com/site/about/guidelines.xhtml

WP2: Biographic Narrative Interviews Procedure

The interviews, ideally, will be conducted in person, with much flexibility around duration and ways in which people can respond (written, verbal, recorded, etc) [34].

These interviews can last up to a maximum of six hours. However, they don't need to be done in one sitting, rather on separate days, or in short bursts of 20 minutes or an hour, by written or oral responses to prompt questions, or a combination of the above; the exact approach will be personalised, based on individual preferences. Since the interview is about the participant's story-telling, it's crucial not to rush them but rather enable their stories to unfold in the way they would like to tell them [35]. Notably, these interviews are not structured, interrogative, inflexible interviews, but the participant is in charge of their story and the way it is told; therefore, these approaches are enjoyed, and do not place the same burden on participants as surveys.

Both peer researchers and research assistants will conduct and analyse interviews. We will ensure that adequate training and support in biographical narrative interviewing is undertaken. We will co-produce a distress protocol during the training to ensure that the relevant processes are in place, and that those conducting the interviews are confident in implementing this protocol.

WP3: Co-Design Procedure

The study will follow the Design Council's Double Diamond approach, which involves four stages: discover, define, develop, and deliver [40]. This process helps to gain a better understanding of the issue at hand and take focused action. The aim of these workshops will be to co-design resources and consider their acceptability, feasibility, barriers to implementation, and mechanisms of effect to improve care experiences of people living with psychosis and MM.

For peer review only - http://bmjopen.bmj.com/site/about/guidelines.xhtml

- Discover

This phase allows a comprehensive understanding of the issue by engaging with those directly impacted by it. This will be undertaken in WP 1 (PV) and 2 (BNI). The outputs from WP 1 and 2 will be analysed and curated to be presented to the core group in a series of priority setting meetings (3-6 of these) to identify key ‘touch points’ that characterise positive and negative experiences.

- Define and Develop

The information gathered in the Discover phase will be used to define the issue. A range of people will be consulted for their views about the problem and solutions will be co-designed. To establish trust, the same key participants - those who have lived experience of the complex phenomenon being examined, as well as those involved in priority setting - will be involved in these phases. The process will entail eight meetings, each lasting two hours.

There will be a series of group meetings, both in-person and online. The initial meetings will involve sharing preliminary ideas and experiences among the core members, followed by identifying patterns and priorities. Once the core group has established their objectives and priorities, they will present these to the wider group for final problem-solving and action-planning. This approach aligns with the psychosocial process employed in Experience-Based Co-Design, which fosters a sense of psychological safety within a group who share similar perspectives, when people are first expressing ideas and exploring how they feel about them.

- Deliver

For peer review only - http://bmjopen.bmj.com/site/about/guidelines.xhtml

The resources co-designed in the design and develop phases will be implemented and improved to adjust to real world situations. This phase will be located in work package 4 as part of the acceptability and feasibility work and a process evaluation of resources within sites. Selection criteria for co-design workshops is presented in Table 2.

Table 2: Experience based co-design workshops selection criteria

**Inclusion**	**Exclusion**
• People with lived experience of receiving care or delivering care in the relevant systems, and people with the power to make decisions and implement changes in the system.	• No lived experience of psychosis or MM or working with people with psychosis or MM.
• Willing and able to provide consent.	

WP4: Evaluation of readiness for Implementation, Acceptability and Feasibility

Phase 1: Baseline ESurvey:

This will be conducted at T1 (before WP1), T2 (after WP2) and T3 (after WP3). At the beginning of the research (T1) we will survey each of the participating regions, including key decision making stakeholders across all sectors in an ICS to establish: plans for integration of care and services; existing work or planned work on psychosis and MM; the priority given to psychosis and multimorbidity, and the relevance of ethnicity in their work; plans over the coming 1,3,5 years; networks of importance and key leaders in the localities that we should contact; invitation to participate in an ongoing network including dissemination of the research and innovations and sharing

For peer review only - http://bmjopen.bmj.com/site/about/guidelines.xhtml

best practice. The survey will be short, 5-10 questions with likert scales and open text boxes. At T2 and T3 the survey will be repeated. The objective is to look at the broad brushstrokes in relation to any shifts in priorities and to sensitise the research team towards the process evaluation.

We will survey up to 120 ICS leaders and managers and practitioners in participating localities as possible, setting a minimum of 10 and up to 30 key stakeholders identified with our co-investigators and project partners from each locality. We will repeat this after WP2, and after WP3, to evaluate receptivity to implement innovations, any changes in the action plans on psychosis and MM in ethnically diverse samples. We anticipate participatory work itself has an impact, so we will be able to gauge to what extent this happens, and variations over time in a descriptive way to inform an assessment of readiness to change and barriers and facilitators. Participants may be invited back for the process evaluation in the last six months of the project after sharing emergent findings.

Phase 2: Process evaluation

A process evaluation will be conducted using the Normalisation Process Theory (NPT) [41]. NPT is a conceptual framework based on four generative mechanisms that underpin the implementation of complex interventions: coherence (what is the work to be done?); cognitive participation (participants have to buy-in to the work); collective action (what work has to be done to enact and enable new practices?); and reflexive monitoring (what work can be done to help appraise new practices?). We will use in-depth semi-structured qualitative interviews with 20-25 professional participants, including decision-makers, commissioners, and health professionals who have used the co-designed resources in hospital, primary, and community care settings. We will also interview up to 20-25 patients and informal carers. Selection criteria for process

For peer review only - http://bmjopen.bmj.com/site/about/guidelines.xhtml

evaluation is presented in Table 3. Patients and carers will be interviewed separately by default, but may choose to be interviewed together. In such cases, interviews will be conducted in a dyadic fashion. Patient participants will be purposively sampled for maximum variation in relation to age, recruitment site, gender, socio-economic background, and mental health status. Furthermore, Health and social care professionals will be surveyed (n=120) using the NoMAD instrument to gather data on implementation practices and structures [42]. The survey questions will be tailored to identify the potential impact of the co-designed resources and whether they will be integrated into routine usage. This will help in determining the usefulness of the resources and inform their future use.

Table 3: Process evaluation survey and interviews' selection criteria

Inclusion	Exclusion
**For survey**	
• Participants will be healthcare professionals working in primary care practices, secondary care mental health services, and/or employees of Integrated Care Systems in clinical and non-clinical roles.	• Those in non-professional or non-NHS facing job roles will be excluded from the survey.
• Willing and able to provide consent.	
**For interviews**	
• Eligibility criteria for patient participants will be the same as WP1.	• Exclusion criteria for patient participants will be the same as WP1.

For peer review only - http://bmjopen.bmj.com/site/about/guidelines.xhtml

• Eligibility criteria for carers will be the same as WP1.	• Exclusion criteria for patient participants will be the same as WP1.
• Health care professionals will be eligible if they are employed in primary care or secondary care mental health services in the same geographic footprint of participating ICSs.	• Healthcare professionals working outside the participating ICS case study regions will be excluded.

For peer review only

WP5: Economic evaluation

Data collected from WP1-3 will be used for economic evaluation. We will ask patient participants to provide information about their experiences of healthcare services before and after their diagnosis of psychosis. In WP1 during the second workshop we will ask participants to provide a 'road map' or 'life-line' to help them recall events. We will use the theoretical model established by Andersen and Newman to investigate the experience of the care system among patient participants [43]. This model identifies three key types of factors that could influence the use of healthcare services: predisposing features (e.g. age, sex, ethnicity, previous illnesses), enabling features (e.g. income, access to care, employment), and needs (e.g. symptoms, diagnosis, functioning) (17). Furthermore, participants will be encouraged to indicate any care they would have preferred to receive but did not, as well as suggest reasons or barriers for why this was the case. The removal of these barriers may have cost implications and could also impact outcomes, which we will explore using economic modelling methods.

Analysis

Data across work packages will be collected in the form of photographic images and captions, written or other reflective accounts of experience, such as drawings and

For peer review only - http://bmjopen.bmj.com/site/about/guidelines.xhtml

poetry, biographical narratives and group discussions, surveys. Data will also be collected via qualitative interviews and a baseline survey concerning available services and the NoMAD survey for a process evaluation The Client Service Receipt Inventory (CSRI) will be used to record the use of health and social care services.

WP1 Photovoice will be analysed using a combination of framework analysis [44] and polytextual thematic analysis [45, 46] to both organise and understand the data. The work package will render three levels of data: demographic data, images and captions and transcripts from the discussion of the images amongst participants from the third workshop. The following eight steps of polytextual thematic analysis will be used to analyse the data [46] : Step 1 – Understanding the sample, step 2 – Organising the data, step 3 – Individual case analysis, step 4 – Exploring interactions, step 5- combining the individual and group analyses, step 6 – Theme generation, Step 7 – Polytextual thematic analysis [46].

WP2 Biographical Narrative Interviews will be audio recorded and transcribed verbatim. The transcripts will be imported to MAXQDA. Thematic Analysis [47] will be used to code the data, and from this, themes will be generated. The resulting themes will be considered alongside the analysis from WP1 to look for points of both similarity and contention. Peer researchers will be supported to also code transcripts and will be part of the team discussions to consider the iteration of higher order themes.

WP3 Experience-Based Co-Design workshops will be audio recorded and transcribed verbatim. The purpose is to identify the context and reasons for decisions about co-design, to address indicated and agreed touchpoints. There may also be realisations that some groups are not going to be well served if a particular design or set of priorities are proposed. The narrative accounts will be described to set out such

For peer review only

decisions, rather than needing a formal qualitative analysis process. We will ensure all respective views and positions are represented and the narrowing of options towards consensus is evident. Those participating in the co-design workshops will be given an opportunity to review the transcripts and summary documents.

WP4 is a process evaluation. which has two phases. Phase 1 is a baseline e survey that will take place at T1 (prior to WP1) T2 (after , WP2)) and T3 (after WP3) and will be analysed descriptively using means, standard deviations and 95% confidence intervals. Inductive content analysis will be used for categorising the free text responses. Responses will be descriptively coded and reviewed to identify representative phrases that are characteristic of or overlap with professional groupings.

Phase 2 of WP4 takes place after WP3 and data will be collected via in depth semi-structured interviews and the NoMAD survey [48]. All interview data will be transcribed verbatim and analysed thematically. An initial thematic coding framework will be developed and checked. Codes in each interview will be examined across individual transcripts as well as the entire data set and allocated to the framework. Using aspects of the constant comparative analysis method broader categories using linked codes will be developed across interviews. Analysis will be guided by items of NPT to structure patients', carers'; and professionals' views about acceptability and implementation readiness of the intervention.

The NoMAD survey tool includes 20 items that reflect the four core NPT constructs and three items to capture data about participants' views about the likelihood that an intervention can become a routine part of their work. The 20-items that compare responses across the four NPT constructs are scored using a 5-point Likert scale to

For peer review only - http://bmjopen.bmj.com/site/about/guidelines.xhtml

indicate the level of agreement, where 1= strongly agree, 3=neutral and 5=strongly disagree. The additional three generic questions about suitability of the intervention for routine practice are scored using a response scale from zero to 10, with 0-not at all; 5= somewhat; and 10 =completely. We will analyse NoMAD responses descriptively, reporting absolute and relative frequencies, along with means and standard deviations and 95% confidence intervals. Where items are negatively phrased responses will be inversely scored. Total scores will not be calculated.

WP5 Health Economics is using The Client Service Receipt Inventory (CRSI) to record the use of health and social care services. These data will be combined with appropriate unit cost information to calculate care costs. Regression models will be used to identify significant demographic and clinical predictors of cost. The potential cost of services not received but perceived as important by participants will also be calculated. Decision modelling will examine the impact of removing care barriers on costs.

Ethics and Dissemination

The study has received a favourable ethics opinion from the Newcastle North Tyneside Research Ethics Committee (Reference number: 23/NE/0143; IRAS 322421). All participants will be provided with detailed information about what participation entails through a participant information sheet, as well as through verbal explanation to ensure full understanding [49]. Only individuals with the capacity to consent will be included in the study, and informed consent will be obtained at each phase of the

For peer review only

research. Participants will be informed that they are free to leave the study at any time without having to provide any reasons. Participants will be assured that they will not face any negative consequences regarding their care. Given that individuals with MM and psychosis from marginalised communities are often more vulnerable, the research team and local systems will take measures to protect, empower, and provide necessary support to participants throughout the research process [16]. This will prevent any potential power imbalances and foster a relationship of trust between researchers and participants. Furthermore, the research methodologies employed seek to minimise stress, provide opportunities for participants to express their artistic abilities, and do not necessitate the verbalisation of emotionally challenging experiences [50]. The confidentiality and anonymity of participants' personal information shall be strictly maintained. The photos and captions will not reveal the identities of the participants unless they make a request for disclosure.

This project will prioritise knowledge exchange and collaborative working. We will take care to disseminate the outputs so the participants do not feel disempowered or lose ownership. To create awareness, we will use online resources such as social media platforms like Twitter, Instagram, and TikTok, as well as a dedicated website and monthly newsletters. Photos and captions taken with WP1 will be shared through exhibitions across local sites to showcase the experiences of individuals with psychosis and MM. The hope is that these shared experiences will inspire service providers and policy makers to work towards improving services. Furthermore, academic publications will be authored along with partners to further the discussions about using creative methodologies and reducing disparities among diverse groups.

For peer review only - http://bmjopen.bmj.com/site/about/guidelines.xhtml

Author contributions

KB is the PI and led the writing of the grant and intellectual work, and prepared revisions, taking feedback from all co-investigators around their specific methodological expertise; UZ, TM, NK contributed to revisions of the protocol before (UZ) and after (TM, NK) securing funding. TM and DJ bring lived experience expertise to design and delivery. Co-investigators' contribute additional methodological expertise: PM-economics; ML-Experience based co-design; DE. PC-evaluation and implementation; JA, FK-sociology and inequalities and qualitative research; FK, RU, RM, KB- photovoice; GH, RU, SG-adversity and life-course epidemiology. RM is a co-investigator who developed the initial protocol for ethics from the funding application.

Funding statement

This study was funded by NIHR HSDR panel (funding reference, NIHR 151887). KB and RM are part supported by Oxford Health NIHR – BRC, and Oxford-TV NIHR-ARC.

Conflicts of interest

None

For peer review only

References

1. Halvorsrud, K., et al., *Identifying evidence of effectiveness in the co-creation of research: a systematic review and meta-analysis of the international healthcare literature.* Journal of Public Health (Oxford, England), 2021. **43**(1): p. 197-208.
2. Das-Munshi, J.D., et al., *Ethnicity and excess mortality in severe mental illness: a cohort study.* The Lancet. Psychiatry, 2017. **4**(5): p. 389-399.
3. Das-Munshi, J., et al., *Type 2 diabetes mellitus in people with severe mental illness: inequalities by ethnicity and age. Cross-sectional analysis of 588 408 records from the UK.* Diabetic medicine, 2017. **34**(7): p. 916-924.
4. Hassan, S., et al., *A qualitative study exploring the barriers and facilitators of implementing a cardiovascular disease risk reducing intervention for people with severe mental illness into primary care contexts across England: the 'PRIMROSE' trial.* BMC Health Services Research, 2020. **20**(1).
5. Excellence, N.I.f.H.a.C. *Multimorbidity: clinical assessment and management*. NICE Guidelines 2016 23rd October 2023]; Available from: https://www.nice.org.uk/guidance/ng56.
6. Owen, R.K., et al., *Effect on life expectancy of temporal sequence in a multimorbidity cluster of psychosis, diabetes, and congestive heart failure among 1·7 million individuals in Wales with 20-year follow-up: a retrospective cohort study using linked data.* The Lancet. Public health, 2023. **8**(7): p. e535-e545.
7. Catalao, R., et al., *Ethnic inequalities in mental and physical multimorbidity in women of reproductive age: a data linkage cohort study.* BMJ open, 2022. **12(7)**.
8. Fonseca de Freitas, D., et al., *Ethnic inequities in multimorbidity among people with psychosis: a retrospective cohort study.* Epidemiology and psychiatric sciences, 2022. **31**: p. e52-e52.
9. England, N. *Improving physical Healthcare for people living with severe mental illness in primary care: Guidance for CCG's*. 2018 23rd October 2023]; Available from: https://www.england.nhs.uk/publication/improving-physical-healthcare-for-people-living-with-severe-mental-illness-smi-in-primary-care-guidance-for-ccgs/.
10. Zahid, U., et al., *Ethnic inequality, multimorbidity and psychosis: can a syndemic framework resolve disputed evidence?* NPJ schizophrenia, 2023. **9**(1): p. 37-37.
11. Singer, M.P., et al., *Syndemics and the biosocial conception of health.* The Lancet (British edition), 2017. **389**(10072): p. 941-950.
12. Bhui, B.K., *Impulsivity, Attachment, and Relational Psychopathology.* British journal of psychiatry, 2018. **213**(3): p. 570-570.
13. Coid, J., et al., *Ethnic disparities in psychotic experiences explained by area-level syndemic effects.* The British Journal of Psychiatry, 2020. **217**: p. 555-561.
14. Hackett, R.A., et al., *Racial discrimination and health: a prospective study of ethnic minorities in the United Kingdom.* BMC public health, 2020. **20**(1): p. 1652-1652.
15. Pefoyo, A.J., et al., *The increasing burden and complexity of multimorbidity.* BMC Public Health, 2015. **15**: p. 415.
16. Willadsen, T.G., et al., *Symptom burden in multimorbidity: a population-based combined questionnaire and registry study from Denmark.* BMJ Open, 2021. **11**(4): p. e041877.
17. Hounkpatin, H.O., et al., *Change in treatment burden among people with multimorbidity: a follow-up survey.* British Journal of General Practice, 2022.
18. Rodgers, M., et al., *Integrated care to address the physical health needs of people with severe mental illness: A mapping review of the recent evidence on barriers, facilitators and evaluations.* International journal of integrated care, 2018. **18**(1): p. 9-9.
19. Whitty, C.J.M. and F.M. Watt, *Map clusters of diseases to tackle multimorbidity.* Nature (London), 2020. **579**(7800): p. 494-496.

For peer review only - http://bmjopen.bmj.com/site/about/guidelines.xhtml

20. Langan, J., S.W. Mercer, and D.J. Smith, *Multimorbidity and mental health: can psychiatry rise to the challenge?* British journal of psychiatry, 2013. **202**(6): p. 391-393.
21. Parker, S.G., et al., *Priorities for research in multiple conditions in later life (multi-morbidity): findings from a James Lind Alliance Priority Setting Partnership.* Age and ageing, 2019. **48**(3): p. 401-406.
22. Wang, C. and M.A. Burris, *Photovoice: Concept, Methodology, and Use for Participatory Needs Assessment.* Health Education & Behavior, 1997. **24**(3): p. 369-387.
23. Council, T.D. *The Double Diamond*. 2023; Available from: https://www.designcouncil.org.uk/our-resources/framework-for-innovation/.
24. Chen, Y.H., M. Karimi, and M. Rutten-van Molken, *The disease burden of multimorbidity and its interaction with educational level.* PLoS One, 2020. **15**(12): p. e0243275.
25. Egid, B.R., et al., *'You want to deal with power while riding on power': global perspectives on power in participatory health research and co-production approaches.* Bmj Global Health, 2021. **6**(11).
26. Bailey, J., et al., *Getting at Equality: Research Methods Informed by the Lessons of Intersectionality.* International Journal of Qualitative Methods, 2019. **18**.
27. Moll, S., et al., *Are you really doing 'codesign'? Critical reflections when working with vulnerable populations.* Bmj Open, 2020. **10**(11).
28. Sutton-Brown, C.A., *Photovoice: A Methodological Guide.* Photography and Culture, 2014. **7**(2): p. 169-185.
29. Budig, K., et al., *Photovoice and empowerment: evaluating the transformative potential of a participatory action research project.* Bmc Public Health, 2018. **18**.
30. Nykiforuk, C.I.J., H. Vallianatos, and L.M. Nieuwendyk, *Photovoice as a Method for Revealing Community Perceptions of the Built and Social Environment.* International Journal of Qualitative Methods, 2011. **10**(2): p. 103-124.
31. Jurkowski, J.M., *Photovoice as participatory action research tool for engaging people with intellectual disabilities in research and program development.* Intellectual and Developmental Disabilities, 2008. **46**(1): p. 1-11.
32. Campbell, R.B., et al., *The challenges of managing diabetes while homeless: a qualitative study using photovoice methodology.* Canadian Medical Association Journal, 2021. **193**(27): p. E1034-E1041.
33. Bennett, B., et al., *The Gaataa'aabing Visual Research Method: A Culturally Safe Anishinaabek Transformation of Photovoice.* International Journal of Qualitative Methods, 2019. **18**.
34. Wengraf, T., *Qualitative Research Interviewing*. First ed. 2001: SAGE Publications Ltd.
35. Chamberlayne P, B.J., Wengraf T, *The Turn to Biographical Methods in Social Science: Comparative Issues and Examples.*: London: Routledge.
36. Brooks, E. and R. Dallos, *Exploring young women's understandings of the development of difficulties: a narrative biographical analysis.* Clin Child Psychol Psychiatry, 2009. **14**(1): p. 101-15.
37. Palinkas, L.A., et al., *Purposeful Sampling for Qualitative Data Collection and Analysis in Mixed Method Implementation Research.* Adm Policy Ment Health, 2015. **42**(5): p. 533-44.
38. Brett, J., et al., *A systematic review of the impact of patient and public involvement on service users, researchers and communities.* Patient, 2014. **7**(4): p. 387-95.
39. Mooney, R., et al., *Experience-based investigation and codesign of approaches to prevent and reduce Mental Health Act use (CO-PACT): a protocol.* BMJ Open, 2022. **12**(2): p. e060086.
40. Council, D. *Framework for Innovation* 2005 26/10/2023]; Available from: https://www.designcouncil.org.uk/our-resources/framework-for-innovation/#:~:text=Design%20Council's%20Double%20Diamond%20clearly,focused%20action%20(convergent%20thinking). .

For peer review only

41. Murray, E., et al., *Normalisation process theory: a framework for developing, evaluating and implementing complex interventions.* Bmc Medicine, 2010. **8**.

42. Finch, T.L., et al., *Improving the normalization of complex interventions: part 2-validation of the NoMAD instrument for assessing implementation work based on normalization process theory (NPT).* Bmc Medical Research Methodology, 2018. **18**.

43. Babitsch, B., D. Gohl, and T. von Lengerke, *Re-revisiting Andersen's Behavioral Model of Health Services Use: a systematic review of studies from 1998-2011.* Psychosoc Med, 2012. **9**: p. Doc11.

44. Spencer, L.R., J., Lewis, J. & Dillon, L., *Quality in Qualitative Evaluation: A framework for assessing research evidence*, G.C.S.R.s. Office, Editor. 2003, Crown Copyright.

45. Gleeson, K., *Polytextual thematic analysis for visual data: analysing visual images*, in *A Handbook of Visual Methods in Psychology*, P. Reavey, Editor. 2021, Routledge: Abingdon. p. 536-554.

46. Mooney, R., et al., *Analysing multimodal data that have been collected using photovoice as a research method.* BMJ open, 2023. **13**(4): p. e068289-e068289.

47. Braun, V. and V. Clarke, *Reflecting on reflexive thematic analysis.* Qualitative research in sport, exercise and health, 2019. **11**(4): p. 589-597.

48. May, C., Rapley, T., Mair ,F.S., Treweek, S., Murray, E., Balllini, L., Macfarlane, A. Girling, M. and Finch, T.L. . *Normalization Process Theory On-line Users' Manual, Toolkit and NoMAD instrument.* 2015 23rd October 2023]; Available from: https://normalization-process-theory.northumbria.ac.uk/.

49. Lieberman, J.A., et al., *Ethical principles and practices for research involving human participants with mental illness.* Psychiatric Services, 2006. **57**(4): p. 552-557.

50. Catalani, C. and M. Minkler, *Photovoice: a review of the literature in health and public health.* Health Educ Behav, 2010. **37**(3): p. 424-51.

For peer review only

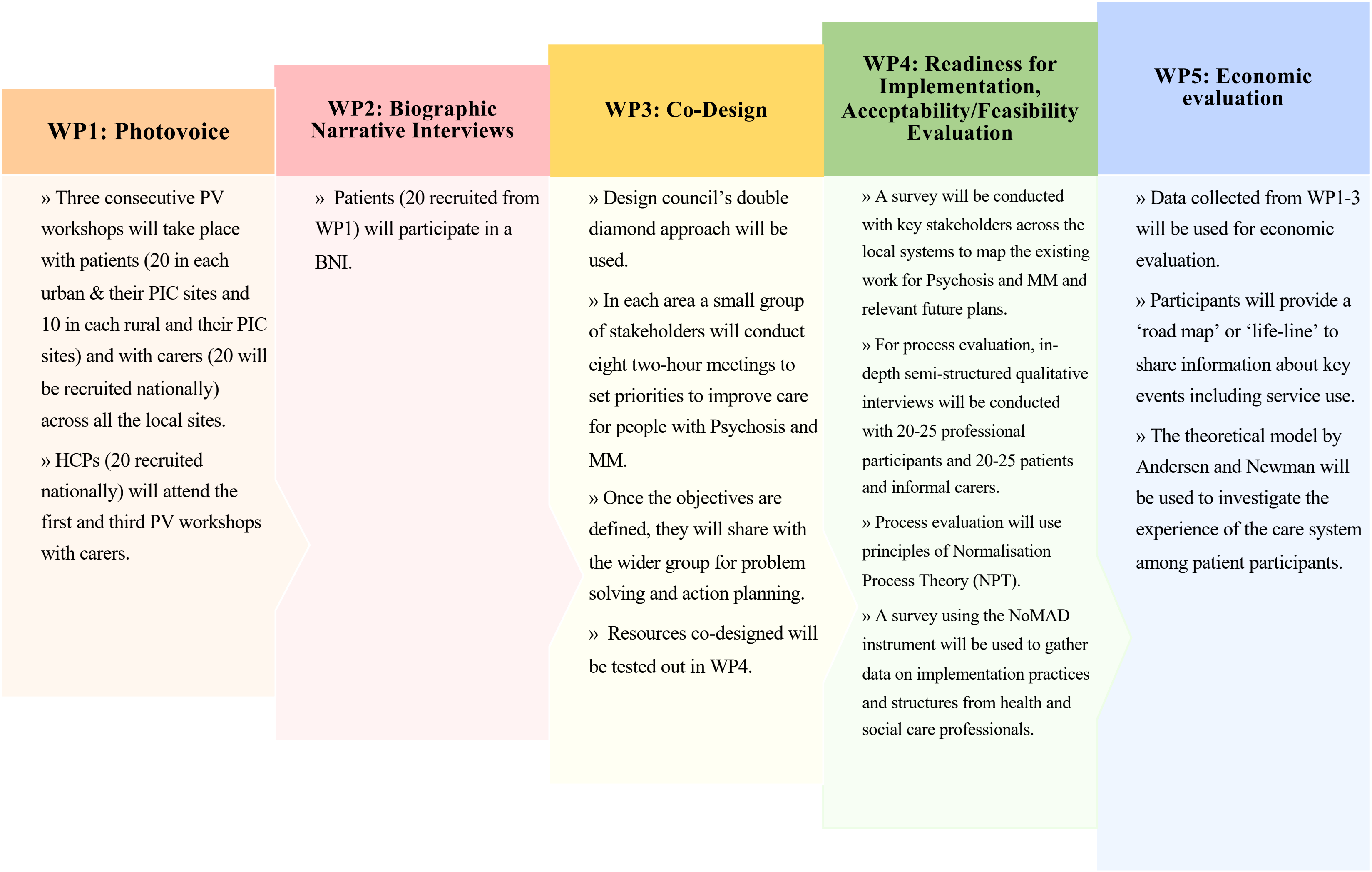

Figure 1: This study will be conducted in seven local systems and three participant identification centres (PIC) situated in London, Birmingham (with a PIC site in Worcestershire), Oxford (with a PIC site in Oxfordshire), Derby, Leicester, Braford (with a PIC site in Lancashire), and Northumberland
