# Supplementary material for: Experience-based Investigation and Co-design of Psychosis Centred Integrated Care Services for Ethnically Diverse People with Multimorbidity (CoPICS): study protocol
Source: BMJ Open. 2024 Feb 28;14(2):e084121. doi: 10.1136/bmjopen-2024-084121 (PMC10910695; doi:10.1136/bmjopen-2024-084121)
Supplement: Reviewer comments [file bmjopen-2024-084121.reviewer_comments.pdf]

PEER REVIEW HISTORY

BMJ Open publishes all reviews undertaken for accepted manuscripts. Reviewers are asked to complete a checklist review form (**http://bmjopen.bmj.com/site/about/resources/checklist.pdf**) and are provided with free text boxes to elaborate on their assessment. These free text comments are reproduced below.

ARTICLE DETAILS

**TITLE (PROVISIONAL)**	Experience-based Investigation and Co-design of Psychosis Centred Integrated Care Services for Ethnically Diverse People with Multimorbidity (CoPICS): Study Protocol.
**AUTHORS**	Bhui , Kamaldeep; Joseph, Doreen; Khan, Nimra; Morrey, Tara; Mooney, Roisin; Zahid, Uzma; Mackay, Tanya; Larkin, Michael; Keating, Frank; McCrone, Paul; Upthegrove, Rachel; Griffiths, Sian Lowri; Edge, Dawn; Coventry, Peter; Arday, Jason; Hosang, Georgina M.

This article was not externally reviewed at BMJ Open. Protocols that have been independently assessed prior to submission to BMJ Open are usually fast-tracked to publication on the grounds that further substantial changes will not be possible. This independent assessment will usually be external, independent review for both a substantial grant award from a non-commercial or government funder and ethics approval.
